# Extracellular Forms of Aβ and Tau from iPSC Models of Alzheimer’s Disease Disrupt Synaptic Plasticity

**DOI:** 10.1016/j.celrep.2018.04.040

**Published:** 2018-05-15

**Authors:** Neng-Wei Hu, Grant T. Corbett, Steven Moore, Igor Klyubin, Tiernan T. O’Malley, Dominic M. Walsh, Frederick J. Livesey, Michael J. Rowan

**Affiliations:** 1 Department of Pharmacology & Therapeutics and Institute of Neuroscience, Trinity College, Dublin 2, Ireland; 2 Department of Physiology and Neurobiology, Zhengzhou University School of Medicine, Zhengzhou 450001, China; 3 Ann Romney Center for Neurologic Diseases, Brigham & Women’s Hospital and Harvard Medical School, Boston, MA 02115, USA; 4 Gurdon Institute and ARUK Stem Cell Research Centre, University of Cambridge, Cambridge CB2 1QN, UK

**Keywords:** Alzheimer’s disease, amyloid β-protein, Down syndrome, extracellular tau, induced pluripotent stem-cell-derived cortical neurons, prion protein, secretome, trisomy 21, dementia

## Abstract

The early stages of Alzheimer’s disease are associated with synaptic dysfunction prior to overt loss of neurons. To identify extracellular molecules that impair synaptic plasticity in the brain, we studied the secretomes of human iPSC-derived neuronal models of Alzheimer’s disease. When introduced into the rat brain, secretomes from human neurons with either a presenilin-1 mutation, amyloid precursor protein duplication, or trisomy of chromosome 21 all strongly inhibit hippocampal long-term potentiation. Synaptic dysfunction caused by presenilin-1 mutant and amyloid precusor protein duplication secretomes is mediated by Aβ peptides, whereas trisomy of chromosome 21 (trisomy 21) neuronal secretomes induce dysfunction through extracellular tau. In all cases, synaptotoxicity is relieved by antibody blockade of cellular prion protein. These data indicate that human models of Alzheimer’s disease generate distinct proteins that converge at the level of cellular prion protein to induce synaptic dysfunction *in vivo*.

## Highlights

• Secretomes of human iPSC-derived models of AD inhibit long-term potentiation *in vivo*
• Familial AD neurons release forms of Aβ that inhibit hippocampal LTP
• Neurons with trisomy of chromosome 21 release a form of tau that blocks LTP
• Blockade of cellular prion protein prevents LTP inhibition by all secretomes

## Graphical Abstract

**Figure:**
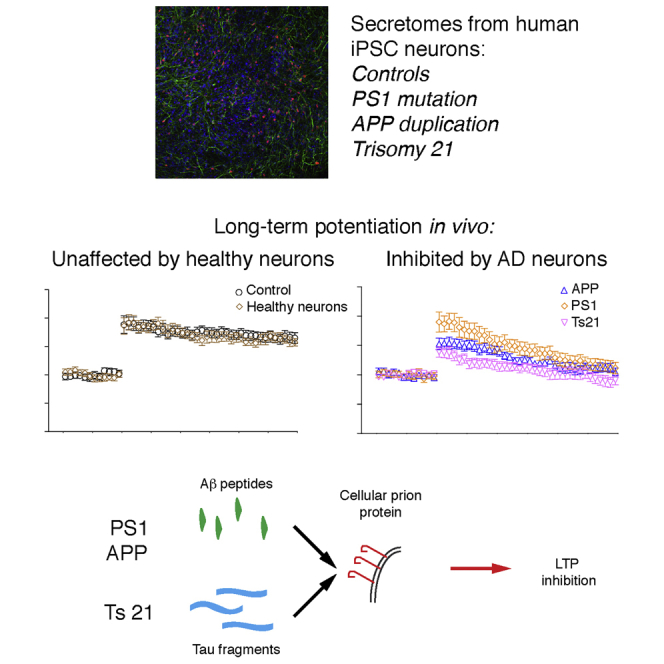

## Teaser

Hu et al. find that human iPSC-derived neurons with autosomal dominant Alzheimer’s disease mutations or trisomy of chromosome 21 release Aβ peptides and tau derivatives that each inhibit a form of long-term synaptic plasticity, LTP, *in vivo*. This disruption occurs via a common pathway that requires cellular prion protein.

## Introduction

In Alzheimer’s disease (AD; Table S1 for a full list of abbreviations), amyloid β-peptides (Aβ) and the microtubule-associated protein tau aggregate and deposit over the course of many decades to produce neuritic plaques and neurofibrillary tangles, respectively (Hyman et al., 2012). While plaques and tangles are pathognomonic for AD, current evidence suggests that soluble extracellular derivatives of amyloid precursor protein (APP) and tau may be the primary cause of synaptic dysfunction and cognitive impairment early in the disease (Goedert, 2016, Spires-Jones and Hyman, 2014).

Deficits in hippocampus-dependent memory are an early feature of AD, and substantial research has focused on the hypothesis that toxic soluble forms of Aβ and tau disrupt hippocampal synaptic memory mechanisms, including long-term potentiation (LTP) (Spires-Jones and Hyman, 2014, Sweatt, 2016). Current approaches testing the effects of Aβ and tau at synapses rely on the study of either synthetic or recombinant proteins (Gómez-Ramos et al., 2006, Walsh et al., 2003) and brain- or cerebrospinal fluid (CSF)-derived material (Fá et al., 2016, Lasagna-Reeves et al., 2012, Yang et al., 2017). While many insights have been gained from this research, controversy has arisen regarding the disease relevance of the forms and concentrations of Aβ and tau used (Benilova et al., 2012, Fá et al., 2016, Hong et al., 2018).

Secretomes of induced pluripotent stem cell (iPSC)-derived neurons carrying causal mutations for AD may better represent the extracellular environment during the early stages of disease and, as such, offer the potential to identify proteins that alter neuronal function (Bright et al., 2015, Goldstein et al., 2015, Moore et al., 2015). We and others previously reported that iPSC-derived neurons harboring mutations in *APP*, presenilin-1 (*PS1*), or trisomy of chromosome 21 (Ts21) exhibit alterations in APP processing, tau metabolism, and tau release (Israel et al., 2012, Moore et al., 2015, Muratore et al., 2014, Shi et al., 2012a, Woodruff et al., 2013, Yagi et al., 2011). To prospectively identify synaptotoxic proteins generated by human neurons, we assessed the ability of secretomes from iPSC-derived models of AD to disrupt LTP when delivered to the hippocampus of live adult rats. We identify two classes of secretomes that powerfully disrupt synaptic plasticity via a common pathway that is mediated by cellular prion protein (PrP).

## Results

### Inhibition of LTP *In Vivo* by the Secretomes of Human Stem Cell Models of AD

**Figure 1:**
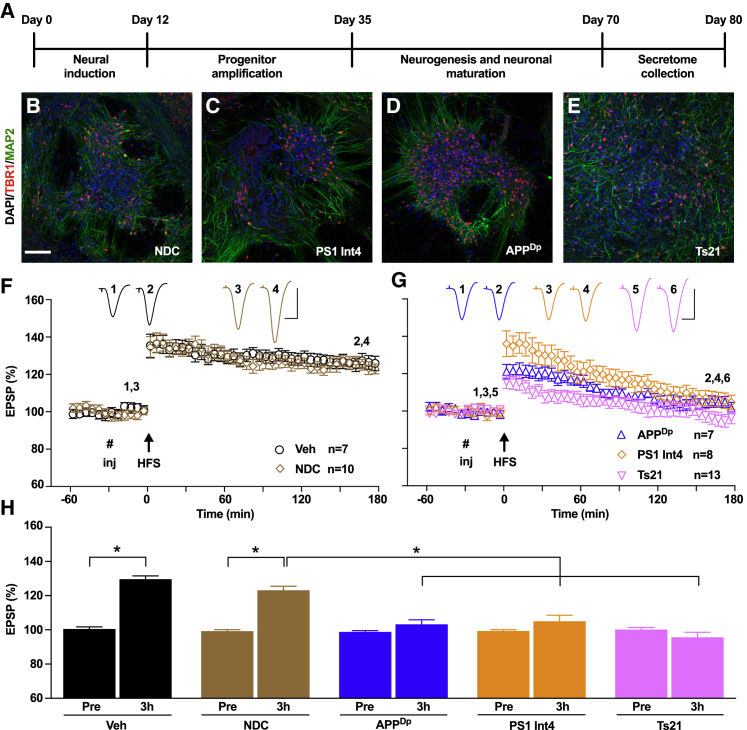
Inhibition of LTP *In Vivo* by Secretomes of Human Stem Cell Models of Alzheimer’s Disease (A) Scheme outlining the generation of cortical cultures and harvesting of secretomes from iPSCs. (B–E) Representative confocal images confirming the differentiation of cortical neurons from NDC (B), PS1 Int4 (C), APP^Dp^ (D), and Ts21 (E) iPSCs of each genotype by the expression of TBR1 (red), a transcription factor expressed in layer 6 glutamatergic neurons, and neuron-specific MAP2 (green) in dendrites at day 80 post-neural induction. Scale bar, 100 μm. (F) The application of high-frequency conditioning stimulation (HFS, arrow) in the hippocampal CA1 area of the anesthetized rat induced a robust and persistent LTP after an intracerebroventricular injection (# inj) of PBS vehicle or NDC secretome (mean ± SEM % pre-HFS baseline at 3 hr: Veh, 129.7 ± 1.8%; NDC, 123.2 ± 2.3%). (G) Injection of secretomes from APP^Dp^ (103.3 ± 2.6%), PS1 Int4 (105.1 ± 3.4%), or Ts21 (95.7 ± 3.0%) neurons completely inhibited LTP at 3 hr post-HFS. The y axis is as shown in (F). (H) Values represent the strength of synaptic transmission before (Pre) and 3 hr after the application of HFS for data in (F) and (G). ^∗^p < 0.05, one-way ANOVA-Sidak and paired t test. n, number of rats. Calibration bars for EPSP traces: vertical, 2 mV; horizontal, 10 ms. See also Figures S1 and S4.

Cortical neurons from non-demented control (NDC) and from three different genetic forms of AD—*PSEN1* L113_I114insT (referred to here as PS1 Int4) (Moore et al., 2015), APP duplication (APP^Dp^) (Israel et al., 2012), and Ts21 (Park et al., 2008)—were generated from iPSCs according to our previously described methods (Shi et al., 2012b). Secretomes were harvested at 48-hr intervals between days 70 and 80 post-neural induction from cultures of cortical glutamatergic neurons and astrocytes (Figures 1A–1E; Figures S1A–S1D). As we previously reported, APP^Dp^ and Ts21 cultures secreted more Aβ than NDC neurons (Figure S1E), and PS1 Int4 neurons exhibited altered Aβ40:Aβ42 and Aβ40:Aβ38 ratios (Figures S1F and S1G) (Moore et al., 2015). Cultures generated from each genotype were confirmed as being cortical in neuronal identity by immunostaining, gene expression, and western blotting, with the only notable difference in cellular composition being an increase in astrocyte number in the Ts21 cultures (Figure S1). Having validated the neuronal composition of the cultures and primary effects of each mutation on Aβ production, we investigated the effects of secretomes on synaptic LTP in anesthetized rats.

Synaptic plasticity was measured in the CA1 area of the dorsal hippocampus following injection of secretomes via a cannula into the lateral ventricle adjacent to the location of the implanted stimulating and recording electrodes under urethane anesthesia. The application of high-frequency conditioning stimulation (HFS) 30 min after the intracerebroventricular (i.c.v.) injection of NDC secretome (10 μL) triggered robust and stable LTP that was indistinguishable from LTP in vehicle-injected rats (Figures 1F and 1H). In contrast, injection of APP^Dp^, PS1 Int4, and Ts21 secretomes all inhibited LTP (Figures 1G and 1H).

### LTP Inhibition by PS1 Int4 and APP^Dp^ Secretomes Is Mediated by Aβ

**Figure 2:**
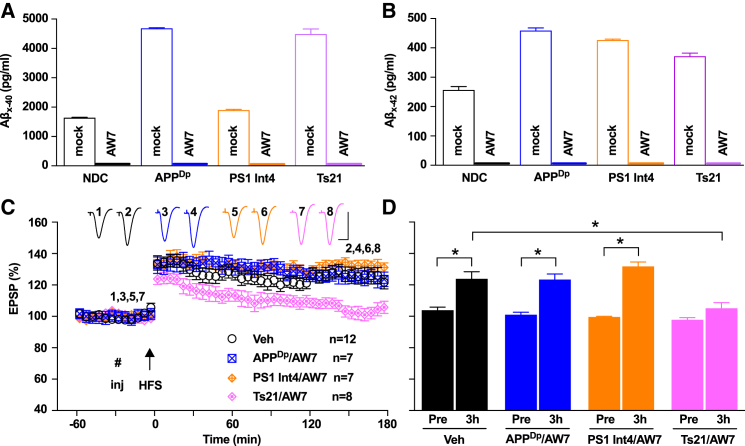
LTP Inhibition by PS1 Int4 or APP^Dp^ Secretomes Is Prevented by Immunodepletion with a Pan-Aβ Polyclonal Antibody (A and B) Neuronal secretomes treated with pre-immune serum (Mock) contain Aβ40 (A) and Aβ42 (B) at levels readily detectable with immunoassays, whereas AW7 immunodepleted secretomes do not contain quantifiable levels of Aβ. (C) Immunodepletion of Aβ peptides with AW7 rescued the inhibition of LTP by APP^Dp^ (APP^Dp^/AW7: 123.4 ± 3.5%) and PS1 Int4 (PS1 Int4/AW7: 131.8 ± 2.8%), but not Ts21 (Ts21/AW7: 105.1 ± 3.7%). Calibration bars for EPSP traces: vertical, 2 mV; horizontal, 10 ms. (D) Values before (Pre) and 3 hr post-HFS. The y axis is as shown in (C). ^∗^p < 0.05, one-way ANOVA-Sidak and paired t test. See also Figures S2 and S4.

The LTP-disrupting secretomes have altered levels or composition of Aβ peptides compared with that of NDC neurons (Figures S1 and S2). Therefore, we tested whether removal of Aβ could prevent LTP inhibition by each secretome. Immunodepletion (ID) of secretomes was performed with the pan-anti-Aβ-antiserum AW7, which recognizes multiple Aβ sequences and aggregation states but not APPsα or Aη peptides (Hong et al., 2018) (see Table S2 for a list of antibodies and their antigens/epitopes). As expected, AW7 ID effectively removed Aβ from neuronal secretomes so that neither Aβ40 nor Aβ42 was detected by ELISA (Figures 2A and 2B). LTP inhibition by APP^Dp^ and PS1 Int4 secretomes was prevented by AW7 ID, confirming that Aβ peptides are responsible for their synaptotoxic effects (Figures 2C and 2D). However, immunodepletion of Aβ peptides did not alter the LTP inhibition mediated by Ts21 secretome (Figures 2C and 2D).

We used size exclusion chromatography (SEC) to fractionate all the secretomes and assayed fractions using Aβ40 and Aβ42 ELISAs and western blotting (Figure S2). Both ELISAs detected a large Aβ monomer peak with negligible amounts of higher-molecular weight species. The APP^Dp^ secretome contained the highest levels of Aβ, and the PS1 Int4 secretome had the highest Aβ42/40 ratio. Otherwise, there were no notable differences between lines (Figures S2A and S2B). Western blotting with 6E10, a monoclonal antibody that recognizes Aβ, N-terminally extended (NTE)-Aβ, Aηα, and APPsα, also failed to reveal differences that could explain why PS1 Int4 and APP^Dp^ secretomes block LTP in an Aβ-dependent manner, whereas the block mediated by Ts21 secretome was independent of Aβ (Figure S2C).

### Extracellular Tau Mediates the Blockade of LTP by the Secretome of Ts21 Neurons

**Figure 3:**
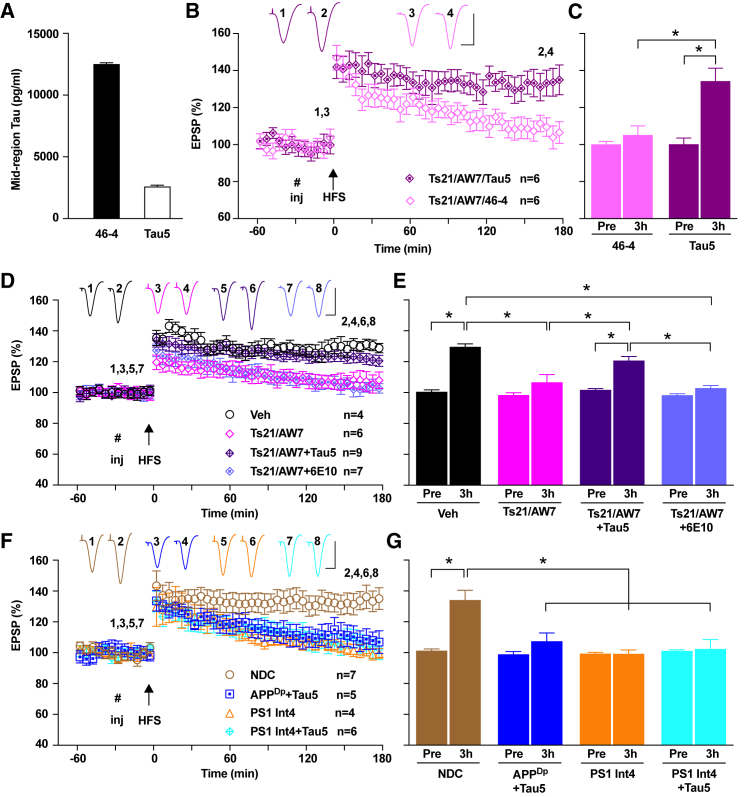
Extracellular Tau Mediates the Blockade of LTP by the Ts21 Secretome (A) Immunodepletion of Ts21 secretome with Tau5 reduced the levels of mid-region containing tau to less than 20% of the original concentration. The monoclonal antibody 46-4 was used as an isotype control. (B and C) Tau immunodepletion with Tau5 prevented the inhibition of LTP by the Ts21 secretome that had previously been immunodepleted with AW7, while mock ID with 46-4 did not (B). Data at 3 hr (Ts21/AW7/Tau5: 134.3 ± 7.0%; Ts21/AW7/46-4: 105.2 ± 4.9%) are summarized in (C). ^∗^p < 0.05, two-way ANOVA RM-Sidak and paired t test. (D and E) Ts21 secretome that had previously been immunodepleted with AW7 inhibited LTP (Ts21/AW7: 106.7 ± 4.9%). Co-injection of Tau5 prevented inhibition of LTP (Ts21/AW7+Tau5: 120.8 ± 2.6%), while an isotype control antibody (6E10, 2.5 μg, i.c.v. injection) did not (Ts21/AW7+6E10: 102.9 ± 1.7%), as summarized in (D) and (E). The y axis is as shown in (D). ^∗^p < 0.05, one-way ANOVA-Sidak and paired t test. (F and G) Stable LTP was induced by HFS 30 min after the i.c.v. injection of NDC secretome. Tau5 (2.5 μg, i.c.v. injection) was co-administered with APP^Dp^ secretome but did not affect the inhibition of LTP (APP^Dp^+Tau5: 107.4 ± 5.4%). LTP was inhibited to a similar extent in animals treated with PS1 Int4 secretome (99.2 ± 2.5%) or co-treated with Tau5 (PS1 Int4+Tau5: 102.4 ± 6.2%), as summarized in (F) and (G). The y axis is as shown in (F). ^∗^p < 0.05, one-way ANOVA-Sidak and paired t test. Calibration bars for EPSP traces: vertical, 2 mV; horizontal, 10 ms. See also Figures S3 and S4.

Our functional analyses demonstrated that the plasticity-disrupting activity in APP^Dp^ and PS1 Int4 secretomes was mediated by a form of Aβ, whereas the activity in the secretome of Ts21 neurons was mediated by a distinct entity. Certain forms of tau are known to inhibit LTP (Fá et al., 2016, Lasagna-Reeves et al., 2012), and tau metabolism is altered in both fAD and Ts21 neurons (Hof et al., 1995, Mondragón-Rodríguez et al., 2014, Portelius et al., 2014). To address the potential involvement of tau, we immunodepleted the Ts21 secretome that previously had been immunodepleted of Aβ (Ts21/AW7), using a mid-region-directed anti-tau antibody, Tau5 (Figure S3A). This reduced the most abundant tau species in the Ts21 secretome by more than 80%, compared with an isotype control antibody (Figure 3A). Ts21 secretome mock-immunodepleted with the isotype control antibody impaired LTP, whereas immunodepletion of tau prevented the inhibition of LTP (Figures 3B and 3C).

To further examine whether synaptotoxic forms of tau were produced by each of the genetic models of AD studied here, an excess of Tau5 (2.5 μg) was co-injected with each secretome. Inhibition of LTP by Ts21/AW7 secretome was prevented by co-injection with Tau5, whereas the anti-Aβ antibody 6E10 (used as an isotype control) had no effect (Figures 3D and 3E). In contrast to results found with Ts21/AW7 secretome, co-injection of Tau5 did not prevent the inhibition of LTP by APP^Dp^ or PS1 Int4 secretomes (Figures 3F and 3G).

To compare the forms of extracellular tau released by neurons of each genotype, we analyzed their secretomes with four ELISAs, which, in combination, can differentiate between full-length (FL), mid-region (MR), N-terminal (NT), and C-terminal (CT) fragments of tau (Figure S3A; Table S3). The relative distribution of extracellular tau was comparable across lines, with MR-containing fragments the most prominent (Table S3). To further resolve different forms of tau, we size-fractionated secretomes from Ts21 and PS1 Int4, which showed that Ts21 secretome contained more SEC early-eluting MR- and CT-reactive material (Figures S3B–S3E). Although less sensitive than ELISA, western blotting detected a broad range of tau fragments and allowed resolution of different-sized species that elute in the same SEC fractions (Figures S3F and S3G). Tau fragments were more abundant in PS1 Int4 than Ts21 secretomes, with the exception of an ∼11-kDa band that was at higher levels in the Ts21 secretome (Figures S3F and S3G, green boxes).

### Independent iPSC Lines Recapitulate the Genotype-Specific Effects of PS1 Int4 and Ts21 Secretomes

We tested secretomes from neurons differentiated from independent non-diseased control (NDC.B), PS1 Int4 (PS1 Int4.B), and Ts21 (Ts21.B) iPSC lines (Figures S4A–S4D) to investigate the reproducibility of our findings. PS1 Int4.B iPSCs were derived from the same donor as in our initial experiments but were reprogrammed independently using a different method. Ts21.B iPSCs were generated from a second independent Ts21 donor. As before, secretomes from healthy control (NDC.B) neurons did not block LTP induction (Figures S4E and S4F). In contrast, PS1 Int4.B secretome blocked LTP, and this activity was relieved by ID of Aβ peptides with AW7 (Figures S4G–S4I), as was found with PS1 Int4 (Figure 2). As with Ts21 secretome (Figure 3), Ts21.B secretome blocked LTP, and this effect was prevented by tau immunodepletion (Figures S4J–S4L).

### Synaptic Dysfunction Induced by PS1 Int4, APP^Dp^, and Ts21 Secretomes Is Dependent on Cellular PrP

**Figure 4:**
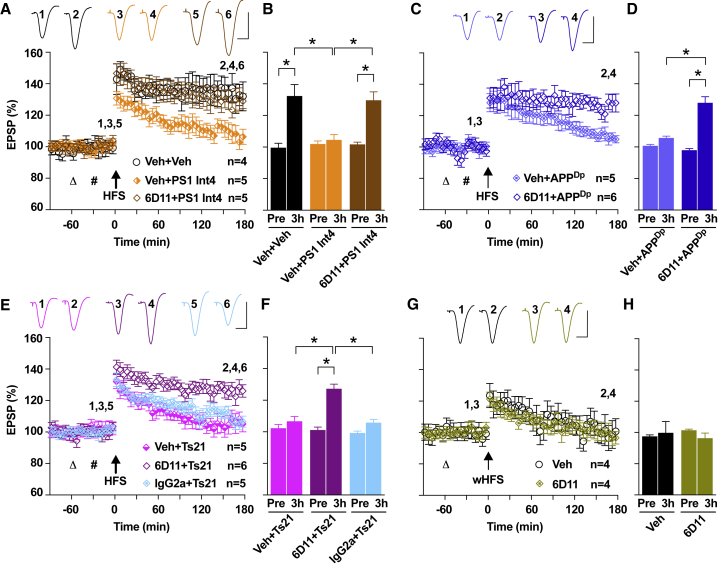
Synaptic Dysfunction Induced by PS1 Int4, APP^Dp^, and Ts21 Secretomes Can Be Relieved by Targeting Cellular PrP (A and B) The inhibition of LTP mediated by the PS1 Int4 secretome (104.6 ± 3.3%) was restored to the level of vehicle controls (132.4 ± 7.2%) by i.c.v. pre-injection (triangle) of 6D11 (6D11+PS1: 129.6 ± 5.3%) (A). Data at 3 hr post-HFS are summarized in (B). The y axis is as shown in (A). ^∗^p < 0.05, one-way ANOVA-Sidak and paired t test. (C and D) Pre-injection of 6D11 also prevented the inhibition of LTP by the APP^Dp^ secretome (C). Data at 3 hr (Veh+APP^Dp^: 105.7 ± 1.1%; 6D11+APP^Dp^: 128.0 ± 3.8%) are summarized in (D). The y axis is as shown in (C). ^∗^p < 0.05, two-way ANOVA RM-Sidak and paired t test. (E and F) The effect of Ts21 secretome (106.7 ± 3.1%) was blocked by 6D11 pre-injection (6D11+Ts21: 127.3 ± 2.9%), but not by an isotype control antibody (IgG2a+Ts21: 105.8 ± 2.1%), as summarized in (E) and (F). The y axis is as shown in (E). ^∗^p < 0.05, one-way ANOVA-Sidak and paired t test. (G and H) A weak conditioning stimulation protocol (wHFS, arrow) induced a decremental LTP (Veh: 99.8 ± 7.2%), and 6D11 alone (96.4 ± 3.2%) did not facilitate this decremental LTP, as summarized in (G) and (H). The y axis is as shown in (G). ^∗^p < 0.05, two-way ANOVA RM-Sidak and paired t test. Calibration bars for EPSP traces: vertical, 2 mV; horizontal, 10 ms.

Given that PrP has been shown to be a mediator of Aβ-induced synaptic dysfunction (Laurén et al., 2009) and has been suggested to act as a sensor for misfolded proteins (Resenberger et al., 2011), we investigated whether PrP was involved in the plasticity defects induced by neural secretomes. Pre-injection of 6D11, an antibody known to prevent Aβ binding to PrP (Laurén et al., 2009), prevented the disruptive effect of PS1 Int4 (Figures 4A and 4B) and APP^Dp^ (Figures 4C and 4D) secretomes on LTP. The inhibition of synaptic plasticity by Ts21 secretome was also prevented by 6D11 but not by an isotype control antibody (Figures 4E and 4F). Furthermore, 6D11 alone did not facilitate a control decremental LTP induced by a weak conditioning stimulation protocol (Figures 4G and 4H). Together, these data indicate that, although the synaptotoxic activity of PS1 Int4, APP^Dp^, and Ts21 secretomes are mediated by distinct proteins, all require PrP to disrupt plasticity.

## Discussion

We report here the use of human iPSC-derived neuronal secretomes and the *in vivo* measurement of LTP to search for extracellular factors that impair synaptic plasticity. Impairment of LTP by APP^Dp^ and PS1 Int4 secretomes is mediated by soluble Aβ, whereas the active entity in Ts21 secretome is a soluble form of tau. The replication of these findings using independent PS1 Int4 and Ts21 secretomes supports the conclusion that several different synaptoxic proteins are released by human stem models of AD.

While the identity and, consequently, the concentration of the toxic species are currently unknown, we infer from our measurements of Aβ and tau that the concentration of active species is likely to be in the low nanomolar/picomolar range. Immediately obvious candidates for the active APP-derived synaptotoxic species in PS1 Int4 and APP^Dp^ secretomes include oligomeric forms of canonical Aβ (Lambert et al., 1998) or NTE-Aβ (Welzel et al., 2014), each of which have been shown to perturb LTP and are recognized by the AW7 antiserum. While we detected these and various other APP derivatives in the secretomes of all lines studied, we discerned no obvious qualitative difference that could explain why only the PS1 Int4 and APP^Dp^ secretomes block LTP in a manner prevented by immunodepletion of Aβ with the AW7 antibody.

Detailed analysis of secretomes using four distinct tau ELISAs, two different western blotting antibodies, and size fractionation revealed a complex mixture of extracellular tau species. Consistent with previous reports, we detected abundant mid-region fragments, lower levels of NT and CT fragments, and very low amounts of FL tau (Bright et al., 2015, Kanmert et al., 2015). While there were no obvious qualitative differences between the forms and levels of tau in Ts21 secretome compared with the others studied here, we did detect two forms of tau that are increased in the Ts21 secretome compared with PS1 Int4. One is a low-abundance fragment that aberrantly elutes from SEC and lacks an intact N terminus, and the second is a more abundant ∼11-kDa fragment that spans at least parts of the mid-region and microtubule-binding-region domains. As yet, it is unclear why a form of tau is the active species in secretomes from Ts21, but not PS1 and APP^Dp^ lines. Trisomy 21 has complex impacts on cellular biology due to widespread perturbation of the proteome (Liu et al., 2017), which may contribute to the differences in extracellular tau production found here.

It has previously been suggested that PrP might serve as a sensor for protein misfolding and that persistent binding of protein aggregates may have adverse effects (Resenberger et al., 2011). Here, we report that plasticity-disrupting activities mediated by extracellular forms of Aβ and tau can be prevented by antibody blockade of PrP. These results support further investigation of synaptotoxic proteins in the secretomes of iPSC-derived neurons and the PrP-dependent mechanism by which they exert their effects. Definitive identification of each synaptotoxic protein will enable further analysis of the cellular mechanisms underlying their ability to inhibit LTP.

## Experimental Procedures

Please see the Supplemental Experimental Procedures and Tables S1 and S2 for full details.

### Study Approval

This research was carried out in accordance with the UK Code of Practice for the Use of Human Stem Cell Lines. The animal care and experimental protocols were carried out in accordance with the approval and oversight of the Irish Health Products Regulatory Authority, Dublin, Ireland.

### Generation of Cortical Cultures and Secretome Collection

Pluripotent stem cells were cultured by standard methods and differentiated to cerebral cortex using established protocols (Shi et al., 2012b). Secretomes were collected at 48-hr intervals between days 70 and 80, after which the cultures were harvested for RNA and protein analysis or fixed for immunohistochemistry. Aβ38, Aβ40, and Aβ42 peptides were analyzed by multiplexed ELISA.

### Secretome Processing and Immunoprecipitation

Secretomes were clarified by centrifugation and dialyzed against artificial cerebrospinal fluid (aCSF) before freezing at −80°C. Immunoprecipitation of Aβ was performed by 2 rounds of 12-hr incubation with AW7 (20 μL) and Protein A Sepharose beads at 4°C. Preimmune serum was used as a control. Tau5 (10 μg) and Protein G Agarose beads were used in 2 rounds of 12-hr incubations at 4°C to immunodeplete tau. The HIV coat protein 1 antibody, 46-4, was used as a control in tau immunoprecipitations.

### SEC, ELISA, and Western Blot

Twelve-milliliter aliquots of ultracentrifuged and dialyzed secretomes were concentrated 10-fold (to 1.2 mL) using Amicon Ultra-15 3-kDa centrifugal filters (Millipore, Billerica, MA, USA) at 4°C. Immediately thereafter, 1 mL concentrate was chromatographed on tandem Superdex 200 Increase-Superdex 75 10/300 GL (GE Healthcare, Marlborough, MA, USA) columns eluted in 50 mM ammonium bicarbonate (pH 8.5) (Amersham Life Sciences, Uppsala, Sweden). One-milliliter fractions were collected and lyophilized for western blot or used for ELISA analysis.

### Electrophysiology

Experiments were carried out on urethane-anesthetized (1.5–1.6 g/kg, intraperitoneally [i.p.]) male Lister Hooded rats (250–350 g), with the exception of 28 similarly sized Wistar rats that were used in the initial studies of the LTP disruptive effect of the Ts21 secretome. Hippocampal LTP was measured by recording field excitatory postsynaptic potentials (EPSPs) from the stratum radiatum of CA1 in response to stimulation of the ipsilateral Schaffer collateral/commissural pathway before and after 200-Hz HFS, as previously described (Hu et al., 2014). Secretomes were injected via cannula into the lateral ventricle of rats 30 min before the induction of synaptic plasticity. Tau5 and 6D11 (both 2.5 μg) were co-injected with secretomes, whereas 6D11 and immunoglobulin G2A (IgG2A) (both 20 μg) were injected 30 min before the application of secretomes to determine the requirement for tau and PrP binding, respectively.

### Data Analysis

All statistical analyses of LTP were conducted in Prism v.6.07 (GraphPad Software, La Jolla, CA, USA). The magnitude of LTP is expressed as the percentage of pre-HFS baseline EPSP amplitude (±SEM). The n refers to the number of animals per group. Control experiments were interleaved randomly throughout. For timeline graphical representation, EPSP amplitudes were grouped into 5-min epochs; for statistical analysis, EPSP amplitudes were grouped into 10-min epochs. One-way ANOVA with Sidak’s multiple comparison test (one-way ANOVA-Sidak) was used for comparisons between groups of three or more. Two-way ANOVA with repeated measures with Sidak’s multiple comparison test (two-way ANOVA RM-Sidak) was used when there were only two groups. Paired t tests were carried out to compare pre- and post-HFS values within groups. A value of p < 0.05 was considered statistically significant.

## Supplemental Information

Document S1. Supplemental Experimental Procedures, Figures S1–S4, and Tables S1–S3

Document S2. Article plus Supplemental Information
