# Supplementary material for: Extracellular Forms of Aβ and Tau from iPSC Models of Alzheimer’s Disease Disrupt Synaptic Plasticity
Source: Cell Rep. 2018 May 15;23(7):1932–8. doi: 10.1016/j.celrep.2018.04.040 (PMC5972225; doi:10.1016/j.celrep.2018.04.040)
Supplement: Document S1. Supplemental Experimental Procedures, Figures S1–S4, and Tables S1–S3 [file mmc1.pdf]

Cell Reports, Volume *23*

Supplemental Information

Extracellular Forms of Aβ and Tau from iPSC Models of Alzheimer's Disease Disrupt Synaptic Plasticity

Neng-Wei Hu, Grant T. Corbett, Steven Moore, Igor Klyubin, Tiernan T. O'Malley, Dominic M. Walsh, Frederick J. Livesey, and Michael J. Rowan

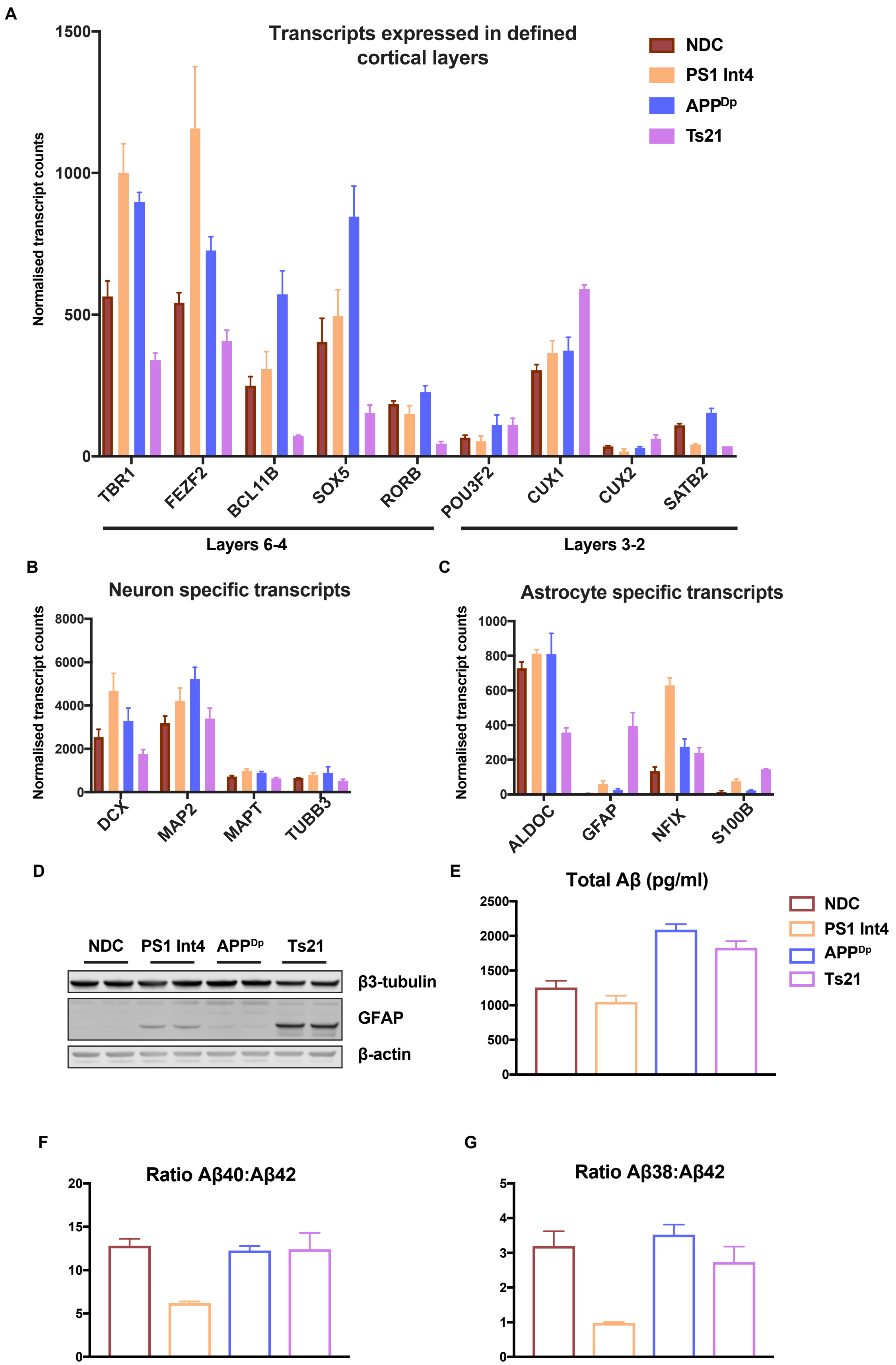

A
Transcripts expressed in defined cortical layers
NDC
PS1 Int4
APPDp
Ts21
Normalised transcript counts
1500
1000
500
0
TBR1
FEZF2
BCL11B
SOX5
RORB
POU3F2
CUX1
CUX2
SATB2
Layers 6-4
Layers 3-2
B
Neuron specific transcripts
Normalised transcript counts
8000
6000
4000
2000
0
DCX
MAP2
MAPT
TUBB3
C
Astrocyte specific transcripts
Normalised transcript counts
1000
800
600
400
200
0
ALDOC
GFAP
NFIX
S100B
D
NDC
PS1 Int4
APPDp
Ts21
β3-tubulin
GFAP
β-actin
E
Total Aβ (pg/ml)
2500
2000
1500
1000
500
0
NDC
PS1 Int4
APPDp
Ts21
F
Ratio Aβ40:Aβ42
20
15
10
5
0
G
Ratio Aβ38:Aβ42
5
4
3
2
1
0

Figure S1. Generation of iPSC-derived human neuronal cultures; Related to Figure 1.

(A) Nanostring analysis of RNA extracted at day 80 post induction. Transcripts associated with deep and upper layer cortical neuronal subtypes were observed in cells derived from each genotype.
(B-C) Transcripts associated with both neuronal and astrocyte identities were expressed by cultures of each genotype, reflecting the composition of the cultures. The data presented in A-C were generated from 3 technical replicates and normalised to the expression levels of a set of 11 housekeeping genes; please see Supplemental Experimental Procedures for more detail. Error bars = S.D.
(D) Western blots of protein extracted at day 80 post induction. PS1 Int4 and APP^{Dp} cultures expressed similar levels of β3-tubulin to NDC. Ts21 cultures exhibited marginally lower expression of β3-tubulin than NDC but had increased expression of the astrocyte marker GFAP, consistent with the transcriptional analysis presented in (B-C). β-actin was used as a loading control for protein levels.
(E) APP^{Dp} and Ts21 neuronal cultures released higher concentrations of total Aβ peptides (Aβ38, Aβ40 and Aβ42) in accordance with their increased APP dosage.
(F-G) PS1 Int4 secretome exhibited a relative increase in Aβ42 compared with Aβ40 (F) and Aβ38 (G), due to the reduction in γ-secretase processivity that we previously described (Moore et al., 2015). The data presented in E-F were generated from 3 technical replicates. Error bars = S.D.

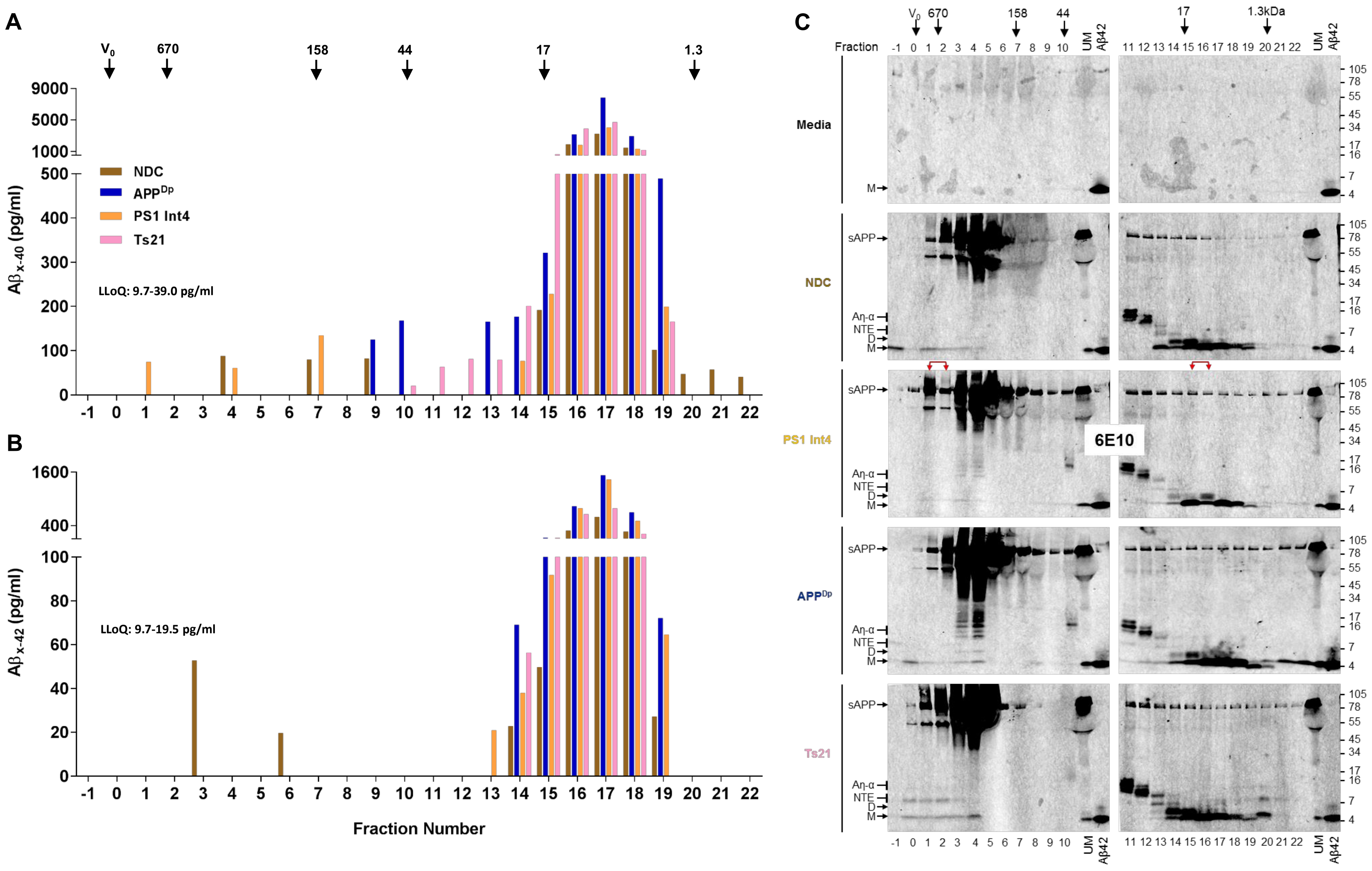
A
B
C
V0
670
158
44
17
1.3
1.3kDa
NDC
APPDp
PS1 Int4
Ts21
LLoQ: 9.7-39.0 pg/ml
LLoQ: 9.7-19.5 pg/ml
Aβx-40 (pg/ml)
Aβx-42 (pg/ml)
Fraction Number
Fraction
Media
6E10
sAPP
Aη-α
NTE
D
M
UM
Aβ42

Figure S2. Secretomes contain an array of APP fragments, including Aβ monomers and dimers, N-terminally extended Aβ and Aη-α; Related to Figure 2.

(A-C) Secretomes from non-demented control (NDC; brown), APP^{Dp} (blue), PS1 Int4 (orange), and Ts21 (pink) neurons were concentrated ten-fold and 1 mL of each concentrate used for SEC. Aliquots of SEC fractions were analyzed for Aβx-40 (A) and Aβx-42 (B) using MSD-based immunoassays. Fraction numbers are indicated on the x-axis and Aβ concentration on the y-axis, and the lower limit of quantitation (LLoQ) of each assay is provided. The elution of globular protein standards (the molecular weight of which are given in kDa) is indicated by downward pointing arrows on the top chromatogram and the void volume, determined from the elution of Blue dextran, is denoted as V_0.

The remainder (0.75 mL) of fractions used in A and B were lyophilized and then analyzed by western blotting. The monoclonal antibody, 6E10, recognizes residues 702-706 of APP_{770}, and detects multiple APP metabolites, including APPsα, Aη-α, N-terminally extended (NTE)-Aβ and Aβ and was used for all blots (C). V_o and elution of globular standards is indicated on the top blot, and SEC fraction numbers are indicated on the top and bottom blots. The secretomes analyzed are indicated on the left. Plain media (Media) was chromatographed, fractions lyophilized and used for western blotting to assess non-specific staining by primary or secondary antibodies. Migration of SDS-PAGE molecular weight standards (in kDa) is indicated on the right of each blot. Arrows indicate Aβ monomer (M), Aβ dimer (D) and sAPP, while dashes indicate NTE-Aβ and Aη-α species. Secretomes that had been concentrated but not chromatographed (designated UM) were mixed with sample buffer and loaded without further manipulation (10 mL). Synthetic Aβ (5 ng) was loaded in the last lane of each blot.

Red arrows above the PS1 Int4 blots in panel C indicate pairs of fractions that were loaded in the reverse order, resulting in exchange of the positions of fractions 1 and 2 on the left blot and fractions 15 and 16 on the right blot.

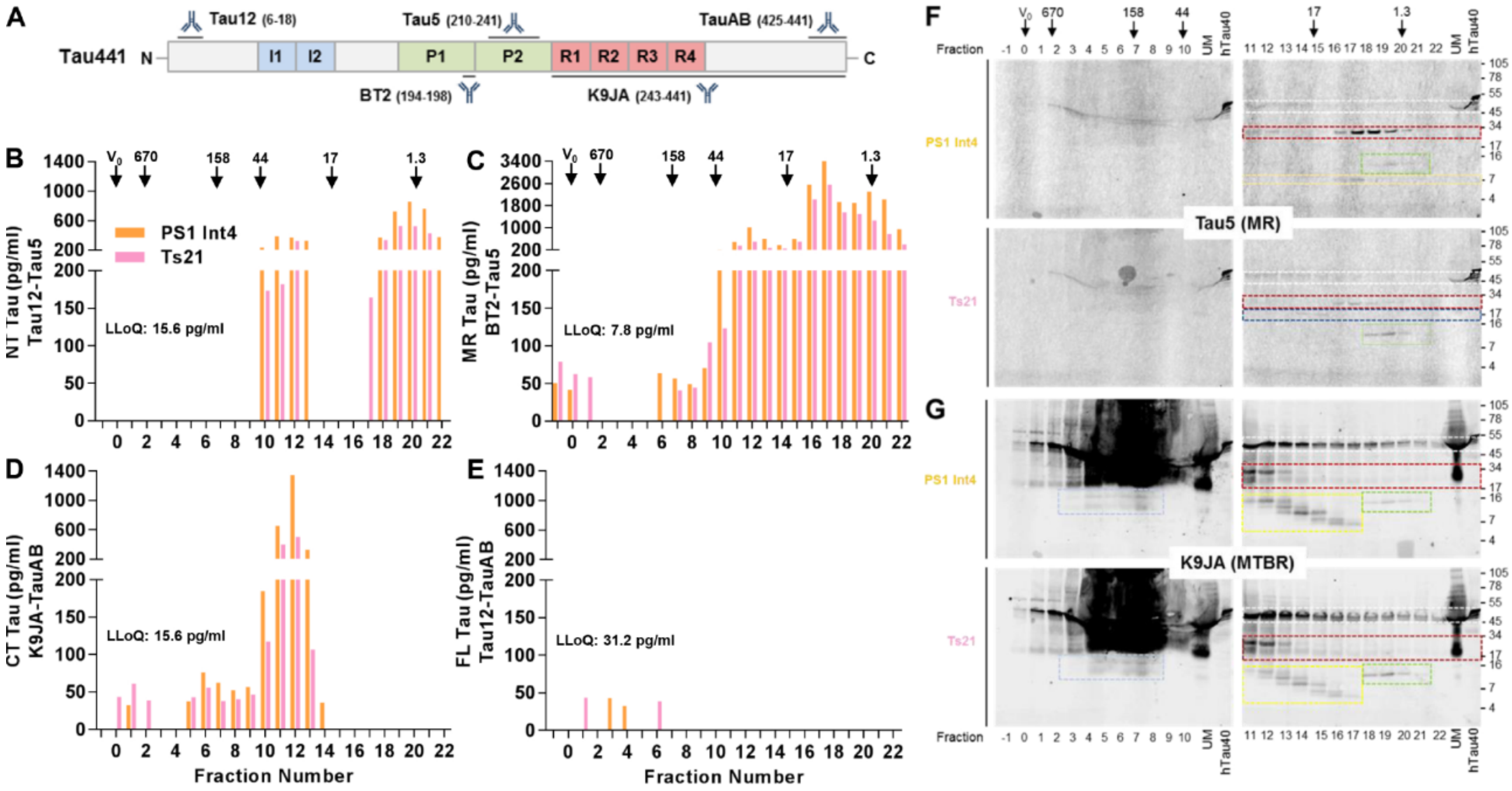

A
Tau441
N
C
Tau12 (6-18)
Tau5 (210-241)
TauAB (425-441)
BT2 (194-198)
K9JA (243-441)
I1
I2
P1
P2
R1
R2
R3
R4
B
NT Tau (pg/ml) Tau12-Tau5
PS1 Int4
Ts21
LLoQ: 15.6 pg/ml
C
MR Tau (pg/ml) BT2-Tau5
LLoQ: 7.8 pg/ml
D
CT Tau (pg/ml) K9JA-TauAB
LLoQ: 15.6 pg/ml
E
FL Tau (pg/ml) Tau12-TauAB
LLoQ: 31.2 pg/ml
Fraction Number
F
G
Fraction
Tau5 (MR)
K9JA (MTBR)
UM
hTau40

Figure S3. Secretomes contain a broad range of truncated tau species, the most abundant of which are mid-region fragments; Related to Figure 3.

(A) Schematic depicting the longest splice isoform of human tau which includes two N-terminal inserts (blue) and four C-terminal repeats (red). Epitopes of the antibodies used for ELISA and western blot are indicated by dark grey lines.

(B-E) Secretomes from PS1 Int4 (orange) and Ts21 (pink) iPSC-derived cortical neurons were concentrated ten-fold and 1 mL of each concentrate used for SEC. Aliquots of SEC fractions were analyzed for tau using 4 different ELISAs. These include N-terminal (NT; B), mid-region (MR; C), C-terminal (CT; D) and full-length (FL; E) assays. The capture and detection antibodies are listed on the y-axes. Fraction numbers are indicated on the x-axis and tau concentration on the y-axis, and the lower limit of quantitation (LLoQ) of each assay is provided. The elution of globular protein standards (the molecular weight of which are given in kDa) is indicated by downward pointing arrows on the top chromatogram and the void volume, determined from the elution of Blue dextran, is denoted as V_0.

The remainder (0.75 mL) of the fractions used in B-E were lyophilized and then analyzed by western blotting using both the mouse antibody, Tau5 (F) and the rabbit antiserum, K9JA (G). The signals for Tau5 and K9JA were detected simultaneously using the two-channel LiCor infrared imaging system and appropriate IR-labeled secondary antibodies. In both F and G, secretomes from PS1 Int4 are shown in the upper panels and Ts21 in lower panels. V_o and elution of globular standards is indicated on the top blot, and SEC fraction numbers are indicated on the top and bottom blots. Migration of SDS-PAGE molecular weight standards (in kDa) is indicated on the right of each blot and colored boxes are used to highlight the position of certain tau species. Unfractionated media (UM) and recombinant human tau 441 (10 ng) were loaded in the last two lanes of each gel.

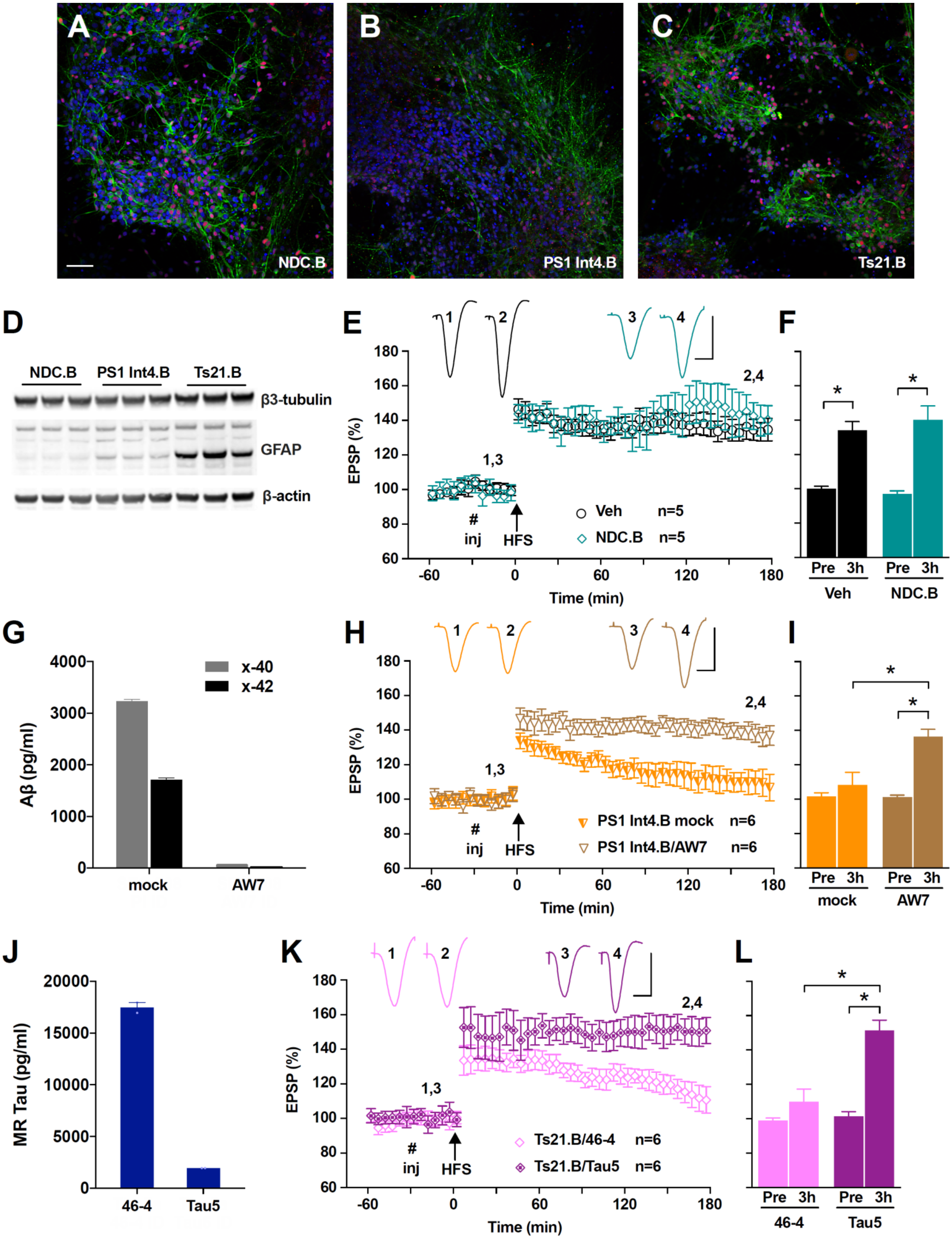
A
NDC.B
B
PS1 Int4.B
C
Ts21.B
D
NDC.B
PS1 Int4.B
Ts21.B
β3-tubulin
GFAP
β-actin
E
EPSP (%)
Time (min)
1,3
2,4
inj
HFS
Veh n=5
NDC.B n=5
F
Pre 3h
Veh
NDC.B
G
Aβ (pg/ml)
x-40
x-42
mock
AW7
H
EPSP (%)
Time (min)
PS1 Int4.B mock n=6
PS1 Int4.B/AW7 n=6
I
mock
AW7
J
MR Tau (pg/ml)
46-4
Tau5
K
EPSP (%)
Time (min)
Ts21.B/46-4 n=6
Ts21.B/Tau5 n=6
L
Pre 3h
46-4
Tau5

Figure S4. Independent iPSC lines recapitulate the genotype-specific effects of PS1 Int4 and Ts21 secretomes; Related to Figures 1, 2 and 3.

(A-C) Representative confocal images confirming the induction of cortical neurons by the expression of TBR1 (red) and MAP2 (green) in neurons of each genotype at day 80 post neural induction. Scale bar: 100 μm.
(D) Western blots of protein extracted at day 80 post-induction. PS1 Int4.B and Ts21.B cultures expressed comparable levels of β3-tubulin to NDC.B controls. GFAP expression was increased in Ts21.B cultures, consistent with the analysis of Ts21 cultures presented in Figure S1.
(E, F) Intracerebroventricular injection (inj, #) of NDC.B secretome 30 min prior to the application of HFS did not affect LTP, with a magnitude (140.1 ± 8.2% at 3 h) comparable to vehicle-injected controls (134.0 ± 5.0%). Data at 3 h post-HFS are summarized in (F).
(G) Immunodepletion of PS1 Int4.B secretome with the pan-Aβ-antiserum AW7 reduced the levels of Aβ40 and Aβ42 to below the limit of detection by ELISA compared with the same secretome treated with control pre-immune serum (Mock).
(H, I) Injection of secretome from PS1 Int4.B treated with control pre-immune serum (mock) completely inhibited LTP at 3 h post-HFS (PS1 Int4.B: 108.2 ± 7.4%). Immunodepletion of Aβ with AW7 prevented the inhibition of LTP by PS1 Int4.B (PS1 Int4.B/AW7: 136.5 ± 4.1%). Data at 3 h post-HFS are summarized in (I).
(J) ID of Ts21.B secretome with the monoclonal antibody Tau5 reduced the levels of mid-region containing tau to less than 12% of the original concentration. The monoclonal antibody 46-4 was used as an isotype control.
(K, L) Immunodepletion of tau with Tau5 ID prevented the inhibition of LTP by the Ts21.B secretome (Ts21.B /Tau5: 151.3 ± 6.1%), while mock ID with 46-4 did not (Ts21.B /46-4: 109.8 ± 7.5%). Data at 3 h post-HFS are summarized in (L).
* $P<0.05$, two-way ANOVA RM-Sidak and paired t. Calibration bars for EPSP traces: vertical, 2 mV; horizontal, 10 ms.

SUPPLEMENTAL EXPERIMENTAL PROCEDURES

Generation of cortical cultures and secretome collection

The iPSC lines used in the this study were NDC (Israel et al., 2012), PS1 Int4 (Moore et al., 2015), APP^{Dp} (Israel et al., 2012) and Ts21 (Park et al., 2008). AD2-1 (here named NDC.B) and SFC808 (here named PS1 Int4.B) iPSCs were obtained from the StemBANCC consortium. Ts21.B iPSCs were generated from a fibroblast biopsy of an individual with Down syndrome using the CytoTune-iPS 2.0 Sendai Reprogramming Kit (ThermoFisher). Human pluripotent stem cells were maintained on Geltrex in Essential 8 media (both ThermoFisher). With minor modifications to account for feeder-free maintenance, directed differentiation of iPSCs to cerebral cortex was performed as previously described (Saurat et al., 2016; Shi et al., 2012a; Shi et al., 2012c). Briefly, multiple iPSC cultures per genotype were induced to form neuroepithelial sheets over the course of 12 days. Cortical progenitors were enriched and expanded between day 12 and 30 before the cultures were dissociated, mixed, aliquoted and frozen in liquid nitrogen. Cortical differentiations were performed by thawing progenitors from each genotype and passaging them into over 30 technical replicates at day 35 post induction. Secretomes of differentiated cultures was collected between day 70 and day 80 post-neural induction at 48 h intervals, aliquoted and stored in protein lo-bind tubes at -80°C.

Immunocytochemistry and imaging

Cultures were fixed at day 80 post neural induction in 4% paraformaldehyde in phosphate buffered saline before immunostaining with TBR1 and MAP2. Nuclei were stained by the addition of DAPI. Imaging was performed on an Olympus FV1000 inverted confocal microscope and processed with Fiji.

Nanostring profiling of cortical cultures

Cultures were harvested at day 80 post neural induction and RNA was extracted using TRIzol (ThermoFisher) before a custom probe set was used to assay transcriptional profile. Hybridization reactions were performed with 50 ng of RNA before post-hybridization and data collection was performed with the nCounter SPRINT Profiler (NanoString Technologies). The data were processed using the nSolver Analysis Software by subtracting the background of the geometric mean of 8 negative controls before normalization to the geometric means of 6 positive

controls and 11 housekeeping genes (CLTC, GAPDH, GUSB, HPRT1, PGK1, PPIA, RPLP1, RPS15A, RPS9, TBP, UXT). This assay was performed on technical triplicates of cultures from each genotype.

Western blot profiling of cortical cultures

Cultures were lysed in RIPA buffer (Sigma) supplemented with cOmplete protease inhibitors and PhosSTOP phosphatase inhibitors (both Roche) at day 80 post neural induction. Western blots were carried out using antibodies to β3-tubulin, GFAP and β-actin and imaged using the LI-COR Odyssey CLx Infrared Imaging System and Image Studio software.

Multiplexed Aβ ELISA

Quantification of Aβ38, Aβ40 and Aβ42 was carried out with multiplexed Meso Scale Discovery assay kits (K15200E) on a Quickplex SQ120 instrument (Meso Scale Discovery, Maryland) using 25µl of cell culture supernatant collected at day 78 post induction.

Clarification and dialysis of secretomes

Secretome was thawed, pooled into 50 mL batches and cleared of cell debris and extracellular vesicles by centrifugation. First, secretome was centrifuged at 200 × *g* and 4°C for 10 min. Then, the upper 97% was recovered (supernatant 1; S1) and centrifuged at 2,000 × *g* and 4°C for 10 min. Next, the upper 97% of this was recovered (S2) and centrifuged at 10,000 × *g* and 4°C for 30 min. Finally, the upper 97% was recovered (S3) and centrifuged at 100,000 × *g* and 4°C for 70 min. The upper 97% of the final supernatant (~44 mL) was dialyzed (using Slide-A-Lyzer™ G2 Dialysis Cassettes, 2K MWCO, ThermoFisher) against artificial cerebrospinal fluid (aCSF; 124 mM NaCl, 2.8 mM KCl, 1.25 mM NaH_2PO_4, 26 mM $NaHCO_3$) to remove bioactive small molecules. Dialysis was performed at 4°C against a 100-fold excess of aCSF with buffer changed three times over a 48 h period. Dialyzed secretome was divided into 1 mL aliquots and either frozen at -80°C or used immediately for immunoprecipitation.

Immunodepletion

Secretome (1 mL) was treated in one of two ways: (i) Immunodepleted (ID) of Aβ by 2 rounds of 12 h incubations with the purified anti-Aβ antiserum, AW7 (20 µL) and Protein A Sepharose beads (PAS; 10 µL; ThermoFisher) at 4°C. (ii) Parallel secretome samples were ‘mock’ ID with purified preimmune serum (PIS; 20 µL) and PAS (10 µL). In each case, samples were cleared of

beads and then both the ID and 'mock' ID samples were incubated with PAS alone to remove previously unbound IgG. To deplete tau species from secretome that had previously been ID with AW7 or PIS, 1 mL aliquots were incubated with the anti-tau monoclonal antibody (mAb) Tau5 (10 µg) and Protein G Agarose beads (PAG) beads (10 µL; Roche) for 2 rounds of 12 h incubations at 4°C. Parallel AW7 or PIS-ID secretome samples were 'mock' ID with 10 µg of the anti-HIV coat protein 1 mAb, 46-4, and PAG (10 µL). Both the Tau5 and 46-4 treated samples were cleared of beads and then both the ID and 'mock' ID samples were incubated with PAG alone to remove previously unbound IgG. ID supernatants were divided into 20 µL aliquots and stored at -80°C until analysis.

Aβx-40/x-42 immunoassays

Analysis of secretomes following clarification, dialysis and immunodepletion utilized highly sensitive, in-house Meso Scale Discovery (MSD) platform-based immunoassays. Unless otherwise indicated, reagents were from Meso Scale (Rockville, MD) and assays were conducted essentially as described previously (Mably et al., 2015). Multi-Array® 96-well small-spot black microplates were coated with 3 µg/mL of monoclonal antibody 266 (Table S2) in tris-buffered saline (TBS) and incubated at room temperature (RT) for 18 h. Unoccupied binding sites were blocked in 150 µL 5% Blocker A in TBS containing 0.05% Tween 20 (TBST) and agitated at 400 rpm for 1 h at 22°C. Plates were then washed three times with 150 µL TBST before samples (diluted 1:1 with 1% Blocker A in TBST) and standards were applied in triplicate and agitated at 400 rpm for 2 h at RT. After capture, plates were washed three times with 150 µL TBST and incubated with biotinylated 2G3 and 21F12 antibodies for x-40 and x-42 detection, respectively. Simultaneously, 1 µg/mL of the reporter reagent (SULFO-TAG labeled streptavidin) was added to diluent and incubated at RT with gentle agitation for 2 h. Finally, plates were washed three times with 150 µL TBST before 2 × MSD read buffer (150 µl per well) was applied to allow for electrochemiluminescence detection. A Sector imager was used to measure the intensity of emitted light, thus allowing the quantitative measurement of analytes present in the samples. The LLoQ, determined by calculating the average + 9 standard errors and 100 ± 20% recovery for each standard, was 39.06 pg/mL and 9.76 pg/mL for x-40 and x-42 assays, respectively.

Tau enzyme-linked immunosorbent assays (ELISAs)

Assays for mid-region tau, N-terminal (NT), C-terminal tau (CT1 and CT2) and full-length (FL) tau were performed using the same procedure but with different combinations of capture and detection antibodies (Kanmert et al., 2015). The antibodies employed, their epitopes, the tau fragment(s) recognized and LLoQs are described in (Table S2). For all assays, the capture antibody was coated at 2.5 µg/mL in TBS for 1 h at 37°C and 300 rpm. Plates were then washed three times with 100 µL TBST prior to blocking in 100 µL TBS containing 3% BSA for 2 h at RT and 300 rpm. Plates were washed three times with 100 µL TBST before 25 µL samples (diluted 1:1 in TBS containing 1% BSA) and standards were applied in triplicate and agitated for 16 h at 4°C. Importantly, the same calibration standard (recombinant human tau441) was used for all assays, thus enabling comparison of concentrations detected by different assays. The following day, 25 µL alkaline phosphatase conjugated detection antibodies diluted 1:250 in TBST containing 1% BSA were added directly to the plates without washing and incubated for 1 h at RT and 300 rpm. Finally, plates were washed three times with 100 µL TBST before 50 µL Tropix Sapphire II (Applied Biosystems) detection reagent was added and incubated for 30 min at RT and 300 rpm. Standard curves were fitted to a five-parameter logistic function with $1/Y^2$ weighting using MasterPlex ReaderFit (MiraiBio). LLoQs were calculated as described for the MSD assays and are provided in Fig. 4.

Animals and surgery

Experiments were carried out on urethane-anesthetized male Lister Hooded rats (250-350 g), with the exception of 28 similarly sized Wistar rats that were used in the initial studies of the LTP disruptive effect of the Ts21 secretome. Since Ts21 secretome had the same inhibitory effect in both strains (28 Wistar and 65 Lister Hooded rats) we combined these data. The animals were housed under a 12h light-dark cycle at room temperature (19-22°C). Prior to the surgery, animals were anesthetized with urethane (1.5-1.6 g/kg, i.p.). Lignocaine (10 mg, 1% adrenaline, s.c.) was injected over the area of the skull where electrodes and screws were to be implanted. The body temperature of the rats was maintained at 37-38 °C with a feedback-controlled heating blanket. A stainless-steel cannula (22 gauge, 0.7 mm outer diameter) was implanted above the right lateral ventricle (1mm lateral to the midline and 4 mm below the surface of the dura) for injecting antibody or the secretome. Intracerebroventricular (i.c.v.) injection was made via an internal cannula (28 gauge, 0.36 mm outer diameter). The solutions were injected in a volume up to 20 µL with a 1.5 µL/min speed. The vehicle injection contained phosphate buffered saline. Because there were no differences between the effects of naïve and

preimmune serum ‘mock’ immunodepleted samples, we combined these results. We carried out initial unblind studies followed by blind studies interleaved with unblind vehicle experiments. For analysis we combined these results with the exception of the data presented for the replication lines in Fig. S4, where we only included blind studies. Verification of the placement of cannula was performed postmortem by checking the spread of ink dye after i.c.v. injection.

Electrophysiology

Electrodes were made and implanted as described previously (Hu et al., 2014). Briefly, twisted bipolar stimulating electrodes were constructed from Teflon-coated tungsten wires (50 μm inner core diameter, 75 μm external diameter) and monopolar recording electrodes were constructed from Teflon-coated tungsten wires (75 μm inner core diameter, 112 μm external diameter) separately. Field excitatory postsynaptic potentials (EPSPs) were recorded from the stratum radiatum in the CA1 area of the right hippocampus in response to stimulation of the ipsilateral Schaffer collateral-commissural pathway. Electrode implantation sites were identified using stereotaxic coordinates relative to bregma, with the recording site located 3.4 mm posterior to bregma and 2.5 mm lateral to midline, and stimulating site 4.2 mm posterior to bregma and 3.8 mm lateral to midline. The final placement of electrodes was optimized by using electrophysiological criteria and confirmed via postmortem analysis.

Test EPSPs were evoked by square wave pulses (0.2 ms duration) at a frequency of 0.033 Hz and an intensity that triggered a 50% maximum response. LTP was induced using 200 Hz high frequency stimulation (HFS) consisting of one set of ten trains of twenty pulses (inter-train interval of 2 s). The stimulation intensity was raised to trigger EPSPs of 75% maximum during the HFS. The conditioning stimulation protocol did not elicit any detectible abnormal changes in background EEG, which was recorded from the hippocampus throughout the experiments. In some animals, a weak high frequency stimulation (wHFS, consisting of 10 trains of 10 pulses at 200 Hz with inter-train interval of 2 s) was used to induce decremental LTP.

Size exclusion chromatography and western blot analysis

Twelve milliliter aliquots of ultracentrifuged and dialyzed unconditioned media or secretomes were concentrated 10-fold (to 1.2 mL) using Amicon Ultra-15 3 kDa centrifugal filters (Millipore, Billerica, MA) at 4ºC. Immediately thereafter, 1 mL of concentrate was chromatographed on tandem Superdex 200 Increase - Superdex 75 10/300 GL (GE, Marlborough, MA) columns eluted in 50

mM ammonium bicarbonate (pH 8.5) at a flow-rate of 0.5 mL/min using Pharmacia FPLC system (Amersham Life Sciences, Uppsala, Sweden). One milliliter fractions were collected and 700 µL was lyophilized for western blot. The remaining 300 µL was aliquoted and stored at -80ºC prior to analyses using tau or $A\beta$ immunoassays. Lyophilisates of SEC fractions were resuspended in 15 µL 1x tricine sample buffer, boiled at 100ºC for 10 min and electrophoresed on 10-20% polyacrylamide tricine gels (ThermoFisher, Waltham, MA). Proteins were transferred onto 0.2 □ M nitrocellulose and the filters boiled in 50 mL PBS and then incubated with the following antibodies overnight: 6E10 (1 µg/mL), or Tau5 (1 µg/mL) and K9JA (1 µg/mL). The following day, blots were washed, incubated with infrared-labeled secondary antibodies (1:17,000; LiCor Biosciences, Lincoln, NE) and imaged using a LiCor Odyssey scanner (LiCor Biosciences, Lincoln, NE).

Antibodies

The antibodies used and their sources are described in Table S2.

Data analysis

All statistical analyses of LTP were conducted in Prism v. 6.07 (GraphPad Software Inc., La Jolla, CA). The magnitude of LTP is expressed as the percentage of pre-HFS baseline EPSP amplitude (± SEM). The n refers to the number of animals per group. Control experiments were interleaved randomly throughout. For time-line graphical representation, EPSP amplitudes were grouped into 5-min epochs; for statistical analysis, EPSP amplitudes were grouped into 10-min epochs. One-way ANOVA with Sidak's multiple comparison test (one-way ANOVA-Sidak) was used to compare between groups of three or more. Two-way ANOVA with repeated measures with Sidak's multiple comparison test (two-way ANOVA RM-Sidak) was used when there were only two groups. Paired *t* tests were carried out to compare pre- and post-HFS values within groups. A value of $P < 0.05$ was considered statistically significant.

SUPPLEMENTAL REFERENCES

Carmel, G., Mager, E.M., Binder, L.I., and Kuret, J. (1996). The structural basis of monoclonal antibody Alz50's selectivity for Alzheimer's disease pathology. J Biol Chem *271*, 32789-32795.

Chang, E.A., Beyhan, Z., Yoo, M.S., Siripattarapravat, K., Ko, T., Lookingland, K.J., Madhukar, B.V., and Cibelli, J.B. (2010). Increased cellular turnover in response to fluoxetine in neuronal precursors derived from human embryonic stem cells. Int J Dev Biol *54*, 707-715.

Ghoshal, N., Garcia-Sierra, F., Wuu, J., Leurgans, S., Bennett, D.A., Berry, R.W., and Binder, L.I. (2002). Tau conformational changes correspond to impairments of episodic memory in mild cognitive impairment and Alzheimer's disease. Exp Neurol *177*, 475-493.

Hu, N.W., Nicoll, A.J., Zhang, D., Mably, A.J., O'Malley, T., Purro, S.A., Terry, C., Collinge, J., Walsh, D.M., and Rowan, M.J. (2014). mGlu5 receptors and cellular prion protein mediate amyloid-beta-facilitated synaptic long-term depression in vivo. Nat Commun *5*, 3374.

Israel, M.A., Yuan, S.H., Bardy, C., Reyna, S.M., Mu, Y., Herrera, C., Hefferan, M.P., Van Gorp, S., Nazor, K.L., Boscolo, F.S.*, et al.* (2012). Probing sporadic and familial Alzheimer's disease using induced pluripotent stem cells. Nature *482*, 216-220.

Jiang, P., Lagenaur, C.F., and Narayanan, V. (1999). Integrin-associated protein is a ligand for the P84 neural adhesion molecule. J Biol Chem *274*, 559-562.

Johnson-Wood, K., Lee, M., Motter, R., Hu, K., Gordon, G., Barbour, R., Khan, K., Gordon, M., Tan, H., Games, D.*, et al.* (1997). Amyloid precursor protein processing and A beta42 deposition in a transgenic mouse model of Alzheimer disease. Proc Natl Acad Sci U S A *94*, 1550-1555.

Kanmert, D., Cantlon, A., Muratore, C.R., Jin, M., O'Malley, T.T., Lee, G., Young-Pearse, T.L., Selkoe, D.J., and Walsh, D.M. (2015). C-terminally truncated forms of tau, but not full-length tau or its C-terminal fragments, are released from neurons independently of cell death. J Neurosci *35*, 10851-10865.

Kim, K.S., Miller, D.L., Chen, C.-M.J., Sapienza, V.J., Bai, C., Grundke-Iqbal, I., Currie, J.R., and Wisniewski, H.M. (1988). Production and characterization of monoclonal antibodies reactive to synthetic cerebrovascular amyloid peptide. Neurosci Res Commun *2*, 121-130.

Mably, A.J., Liu, W., Mc Donald, J.M., Dodart, J.C., Bard, F., Lemere, C.A., O'Nuallain, B., and Walsh, D.M. (2015). Anti-Abeta antibodies incapable of reducing cerebral Abeta oligomers fail to attenuate spatial reference memory deficits in J20 mice. Neurobiol Dis *82*, 372-384.

McDonald, J.M., Cairns, N.J., Taylor-Reinwald, L., Holtzman, D., and Walsh, D.M. (2012). The levels of water-soluble and triton-soluble Abeta are increased in Alzheimer's disease brain. Brain Res *1450*, 138-147.

Mercken, M., Vandermeeren, M., Lubke, U., Six, J., Boons, J., Van de Voorde, A., Martin, J.J., and Gheuens, J. (1992). Monoclonal antibodies with selective specificity for Alzheimer Tau are directed against phosphatase-sensitive epitopes. Acta Neuropathol *84*, 265-272.

Moore, S., Evans, L.D., Andersson, T., Portelius, E., Smith, J., Dias, T.B., Saurat, N., McGlade, A., Kirwan, P., Blennow, K*., et al.* (2015). APP metabolism regulates tau proteostasis in human cerebral cortex neurons. Cell Rep *11*, 689-696.

Pankiewicz, J., Prelli, F., Sy, M.S., Kascsak, R.J., Kascsak, R.B., Spinner, D.S., Carp, R.I., Meeker, H.C., Sadowski, M., and Wisniewski, T. (2006). Clearance and prevention of prion infection in cell culture by anti-PrP antibodies. Eur J Neurosci *23*, 2635-2647.

Park, I.H., Arora, N., Huo, H., Maherali, N., Ahfeldt, T., Shimamura, A., Lensch, M.W., Cowan, C., Hochedlinger, K., and Daley, G.Q. (2008). Disease-specific induced pluripotent stem cells. Cell *134*, 877-886.

Saurat, N.G., Livesey, F.J., and Moore, S. (2016). Cortical Differentiation of Human Pluripotent Cells for In Vitro Modeling of Alzheimer's Disease. Methods Mol Biol *1303*, 267-278.

Shi, Y., Kirwan, P., and Livesey, F.J. (2012a). Directed differentiation of human pluripotent stem cells to cerebral cortex neurons and neural networks. Nat Protoc *7*, 1836-1846.

Shi, Y., Kirwan, P., Smith, J., Robinson, H.P., and Livesey, F.J. (2012c). Human cerebral cortex development from pluripotent stem cells to functional excitatory synapses. Nat Neurosci *15*, 477-486.

Wang, Y.P., Biernat, J., Pickhardt, M., Mandelkow, E., and Mandelkow, E.M. (2007). Stepwise proteolysis liberates tau fragments that nucleate the Alzheimer-like aggregation of full-length tau in a neuronal cell model. Proc Natl Acad Sci U S A *104*, 10252-10257.

Supplemental Table S1, Related to Experimental Procedures and main text.
List of Abbreviations

Abbreviation	Meaning
aCSF	artificial cerebrospinal fluid
AD	Alzheimer's disease
APP	amyloid precursor protein
APP^{Dp}	amyloid precursor protein gene duplications
APP^{Dp}.B	amyloid precursor protein gene duplications line B
APPsα	soluble APP-alpha
Aβ	amyloid β-protein
Aη	aη fragment of APP
A$\eta\alpha$	Soluble A$\eta\alpha$ fragment of APP
CT	C-terminal
ELISA	enzyme-linked immunosorbent assay
fAD	familial Alzheimer's disease
GFAP	glial fibrillary acidic protein
FL	full-length
i.c.v.	intracerebroventricular
ID	immunodepleted
iPSC	induced pluripotent stem cell
LTP	long-term potentiation
mAb	monoclonal antibody
NDC	non-demented control
NDC.B	non-demented control line B
MAP2	microtubule-associated protein 2
MR	mid-region
MTBR	microtubule-binding region
NT	N-terminal
NTE-Aβ	N-terminally extended Aβ
PAG	protein G Agarose beads
PAS	protein A Sepharose beads
PIS	preimmune serum
PrP	*prion protein*
PS1 Int4	mutation in *PSEN1* L113_I114insT

PS1 Int4.B	mutation in *PSEN1* L113_I114insT line B
rpm	revolutions per minute
RT	room temperature
SEC	size exclusion chromatography
TBR1	the neuron-specific transcription factor T-box brain 1
TBS	tris-buffered saline
TBST	tris-buffered saline containing 0.05% Tween 20
Ts21	mutation in trisomy of chromosome 21
Ts21.B	mutation in trisomy of chromosome 21 line B

Supplemental Table S2, Related to Experimental Procedures.
Primary antibodies and their antigens, dilutions and sources.

Antibody	Clonality	Antigen/Epitope	Dilution	Source	Reference
AW7	Polyclonal	Pan-Aβ	1:50 (IP)	Walsh Lab	(McDonald et al., 2012)
6E10	Monoclonal	702-706 of APP_{770}	1 μg/ml (WB); (i.c.v. injection)	Biolegend	(Kim et al., 1988)
266	Monoclonal	Aβ13-26	1 μg/ml (ELISA)	Janssen	(Johnson-Wood et al., 1997)
2G3	Monoclonal	Aβ terminating at Val40	1 μg/ml (ELISA)	Janssen	(Johnson-Wood et al., 1997)
21F12	Monoclonal	Aβ terminating at Ile42	1 μg/ml (ELISA)	Janssen	(Johnson-Wood et al., 1997)
Tau12	Monoclonal	Tau 6-18	2.5 μg/ml (ELISA)	Millipore	(Ghoshal et al., 2002)
BT2	Monoclonal	Tau 194-198	2.5 μg/ml (ELISA)	ThermoFisher	(Mercken et al., 1992)
Tau5	Monoclonal	Tau 210-241	1 μg/ml (WB); 1:100 (IP); 2.5 μg/ml (ELISA)	BioLegend	(Carmel et al., 1996)
K9JA	Polyclonal	Tau 243-441	1 μg/ml (WB); 2.5 μg/ml (ELISA)	Dako	(Wang et al., 2007)
TauAB	Monoclonal	Tau 425-441	2.5 μg/ml (ELISA)	MedImmune	Gift from A. Billinton/ M. Perkinton
46-4	Monoclonal	Anti-HIV	1:100 (IP)	ATCC	(Wang et al., 2007)
Anti-TBR1	Polyclonal	TBR1 50-150	1:1500 (ICC)	Abcam	(Shi et al., 2012c)
Anti-MAP2	Polyclonal	Full length MAP2	1:2000 (ICC)	Abcam	(Shi et al., 2012c)
Anti- β3-tubulin	Monoclonal	C-terminus of β3-tubulin	1:1000 (WB)	Biolegend	(Moore et al., 2015)
GFAP	Polyclonal	Full length GFAP	1:1000 (WB)	Abcam	(Chang et al., 2010)
Anti- β-actin	Monoclonal	N-terminus of β-actin	1:10000 (WB)	Sigma	(Moore et al., 2015)
6D11	Monoclonal	PrP 93-109	(i.c.v. injection)	BioLegend	(Pankiewicz et al., 2006)
IgG2a	Monoclonal	N/A	(i.c.v. injection)	BioLegend	(Jiang et al., 1999)

Supplemental Table S3, Related to Figure 3.
Summary of the tau species detected by ELISAs in iPSC-derived cortical neuron secretomes after mock or AW7 immunodepletion.

	NDC		Ts21		APP^{Dp}		PS1 Int4	
	**Mock ID**	**AW7 ID**	**Mock ID**	**AW7 ID**	**Mock ID**	**AW7 ID**	**Mock ID**	**AW7 ID**
**Full-length**	488.3 ± 0.6	266.7 ± 4.7	403.7 ± 7.2	737.1 ± 2.4	700.2 ± 18.8	1081.5 ± 21.9	724.7 ± 14.3	2,085.8 ± 20.2
**N-terminal**	4,574.3 ± 438.3	4,849.7 ± 91.3	1,873.7 ± 430.3	2,406.4 ± 111.3	8,362.5 ± 684.5	9,662.9 ± 288.0	4,101.058 ± 84.7	6,549.2 ± 461.0
**Mid-region**	21,378.0 ± 682.8	21,137.8 ± 582.9	6,956.4 ± 578.6	8,352.7 ± 302.9	44,597.8 ± 28.2	42,967.1 ± 545.8	15,747.4 ± 113.8	16,811.5 ± 479.1
**C-terminal 1**	1,730.8 ±127.8	1,781.5 ± 72.8	1,089.1 ± 8.0	2,103.2 ± 122.6	3,033.7 ± 190.9	3,869.3 ± 119.7	2,170.0 ± 64.5	4,522.3 ± 61.9
**C-terminal 2**	502.4 ± 2.1	351.5 ± 3.7	438.0 ± 0.8	752.9 ± 18.3	631.4 ± 1.5	965.3 ± 5.5	800.1 ± 45.5	1,991.2 ±61.9

Key: Values represent mean ± SD in pg/ml of three technical replicates. ND = not detected. NDC = non-demented control secretome, Ts21 = Trisomy 21 secretome, APP^{Dp} = *APP* duplication secretome, PS1 Int4 = *PSEN1* intron 4 secretome. Mock ID = mock immunodepletion, AW7 ID = immunodepletion with AW7
