# Supplementary material for: Extracellular Forms of Aβ and Tau from iPSC Models of Alzheimer’s Disease Disrupt Synaptic Plasticity
Source: Cell Rep. 2018 May 15;23(7):1932–8. doi: 10.1016/j.celrep.2018.04.040 (PMC5972225; doi:10.1016/j.celrep.2018.04.040)
Supplement: Document S2. Article plus Supplemental Information [file mmc2.pdf]

Cell Reports

Report

Extracellular Forms of Aβ and Tau from iPSC Models of Alzheimer's Disease Disrupt Synaptic Plasticity

Graphical Abstract

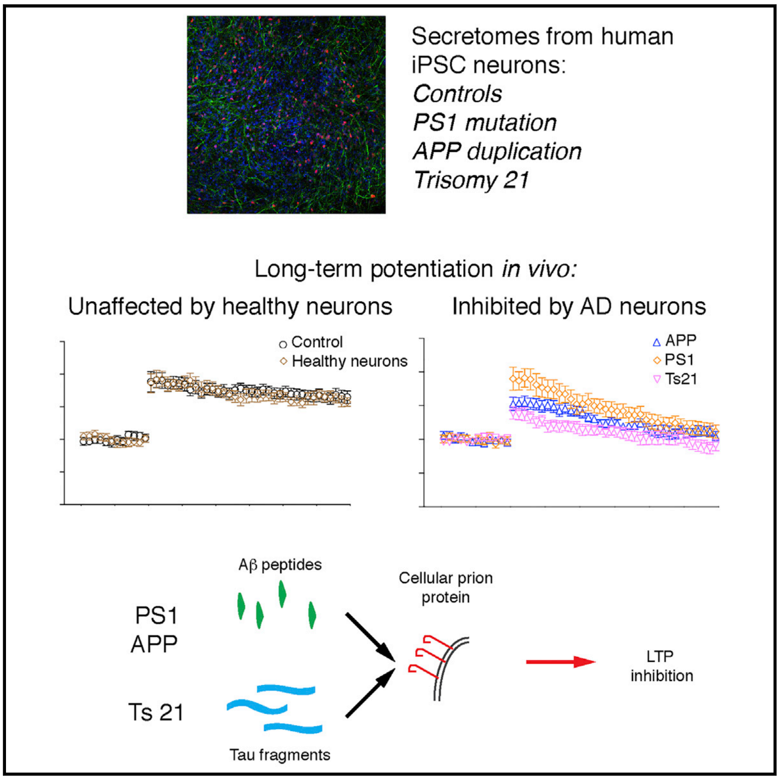

Authors

Neng-Wei Hu, Grant T. Corbett, Steven Moore, ..., Dominic M. Walsh, Frederick J. Livesey, Michael J. Rowan

Correspondence

dwalsh3@bwh.harvard.edu (D.M.W.), rick@gurdon.cam.ac.uk (F.J.L.), mrowan@tcd.ie (M.J.R.)

In Brief

Hu et al. find that human iPSC-derived neurons with autosomal dominant Alzheimer's disease mutations or trisomy of chromosome 21 release Aβ peptides and tau derivatives that each inhibit a form of long-term synaptic plasticity, LTP, *in vivo*. This disruption occurs via a common pathway that requires cellular prion protein.

Highlights

- Secretomes of human iPSC-derived models of AD inhibit long-term potentiation *in vivo*
- Familial AD neurons release forms of Aβ that inhibit hippocampal LTP
- Neurons with trisomy of chromosome 21 release a form of tau that blocks LTP
- Blockade of cellular prion protein prevents LTP inhibition by all secretomes

Hu et al., 2018, Cell Reports *23*, 1932–1938
May 15, 2018 © 2018 The Authors.
https://doi.org/10.1016/j.celrep.2018.04.040

OPEN ACCESS
CellPress

Cell Reports
Report

Extracellular Forms of Aβ and Tau from iPSC Models of Alzheimer's Disease Disrupt Synaptic Plasticity

Neng-Wei Hu,[1,2,5] Grant T. Corbett,[3,5] Steven Moore,[4,5] Igor Klyubin,[1] Tiernan T. O'Malley,[3] Dominic M. Walsh,[3,*] Frederick J. Livesey,[4,6,*] and Michael J. Rowan[1,*]
[1]Department of Pharmacology & Therapeutics and Institute of Neuroscience, Trinity College, Dublin 2, Ireland
[2]Department of Physiology and Neurobiology, Zhengzhou University School of Medicine, Zhengzhou 450001, China
[3]Ann Romney Center for Neurologic Diseases, Brigham & Women's Hospital and Harvard Medical School, Boston, MA 02115, USA
[4]Gurdon Institute and ARUK Stem Cell Research Centre, University of Cambridge, Cambridge CB2 1QN, UK
[5]These authors contributed equally
[6]Lead Contact
*Correspondence: dwalsh3@bwh.harvard.edu (D.M.W.), rick@gurdon.cam.ac.uk (F.J.L.), mrowan@tcd.ie (M.J.R.)
https://doi.org/10.1016/j.celrep.2018.04.040

SUMMARY

The early stages of Alzheimer's disease are associated with synaptic dysfunction prior to overt loss of neurons. To identify extracellular molecules that impair synaptic plasticity in the brain, we studied the secretomes of human iPSC-derived neuronal models of Alzheimer's disease. When introduced into the rat brain, secretomes from human neurons with either a presenilin-1 mutation, amyloid precursor protein duplication, or trisomy of chromosome 21 all strongly inhibit hippocampal long-term potentiation. Synaptic dysfunction caused by presenilin-1 mutant and amyloid precusor protein duplication secretomes is mediated by Aβ peptides, whereas trisomy of chromosome 21 (trisomy 21) neuronal secretomes induce dysfunction through extracellular tau. In all cases, synaptotoxicity is relieved by antibody blockade of cellular prion protein. These data indicate that human models of Alzheimer's disease generate distinct proteins that converge at the level of cellular prion protein to induce synaptic dysfunction *in vivo*.

INTRODUCTION

In Alzheimer's disease (AD; Table S1 for a full list of abbreviations), amyloid β-peptides (Aβ) and the microtubule-associated protein tau aggregate and deposit over the course of many decades to produce neuritic plaques and neurofibrillary tangles, respectively (Hyman et al., 2012). While plaques and tangles are pathognomonic for AD, current evidence suggests that soluble extracellular derivatives of amyloid precursor protein (APP) and tau may be the primary cause of synaptic dysfunction and cognitive impairment early in the disease (Goedert, 2016; Spires-Jones and Hyman, 2014).

Deficits in hippocampus-dependent memory are an early feature of AD, and substantial research has focused on the hypothesis that toxic soluble forms of Aβ and tau disrupt hippocampal synaptic memory mechanisms, including long-term potentiation (LTP) (Spires-Jones and Hyman, 2014; Sweatt, 2016). Current approaches testing the effects of Aβ and tau at synapses rely on the study of either synthetic or recombinant proteins (Gómez-Ramos et al., 2006; Walsh et al., 2003) and brain- or cerebrospinal fluid (CSF)-derived material (Fá et al., 2016; Lasagna-Reeves et al., 2012; Yang et al., 2017). While many insights have been gained from this research, controversy has arisen regarding the disease relevance of the forms and concentrations of Aβ and tau used (Benilova et al., 2012; Fá et al., 2016; Hong et al., 2018).

Secretomes of induced pluripotent stem cell (iPSC)-derived neurons carrying causal mutations for AD may better represent the extracellular environment during the early stages of disease and, as such, offer the potential to identify proteins that alter neuronal function (Bright et al., 2015; Goldstein et al., 2015; Moore et al., 2015). We and others previously reported that iPSC-derived neurons harboring mutations in *APP*, presenilin-1 (*PS1*), or trisomy of chromosome 21 (Ts21) exhibit alterations in APP processing, tau metabolism, and tau release (Israel et al., 2012; Moore et al., 2015; Muratore et al., 2014; Shi et al., 2012a; Woodruff et al., 2013; Yagi et al., 2011). To prospectively identify synaptotoxic proteins generated by human neurons, we assessed the ability of secretomes from iPSC-derived models of AD to disrupt LTP when delivered to the hippocampus of live adult rats. We identify two classes of secretomes that powerfully disrupt synaptic plasticity via a common pathway that is mediated by cellular prion protein (PrP).

RESULTS

Inhibition of LTP *In Vivo* by the Secretomes of Human Stem Cell Models of AD

Cortical neurons from non-demented control (NDC) and from three different genetic forms of AD—*PSEN1* L113_I114insT (referred to here as PS1 Int4) (Moore et al., 2015), APP

 © 2018 The Authors.
This is an open access article under the CC BY license (http://creativecommons.org/licenses/by/4.0/).

OPEN ACCESS
CellPress

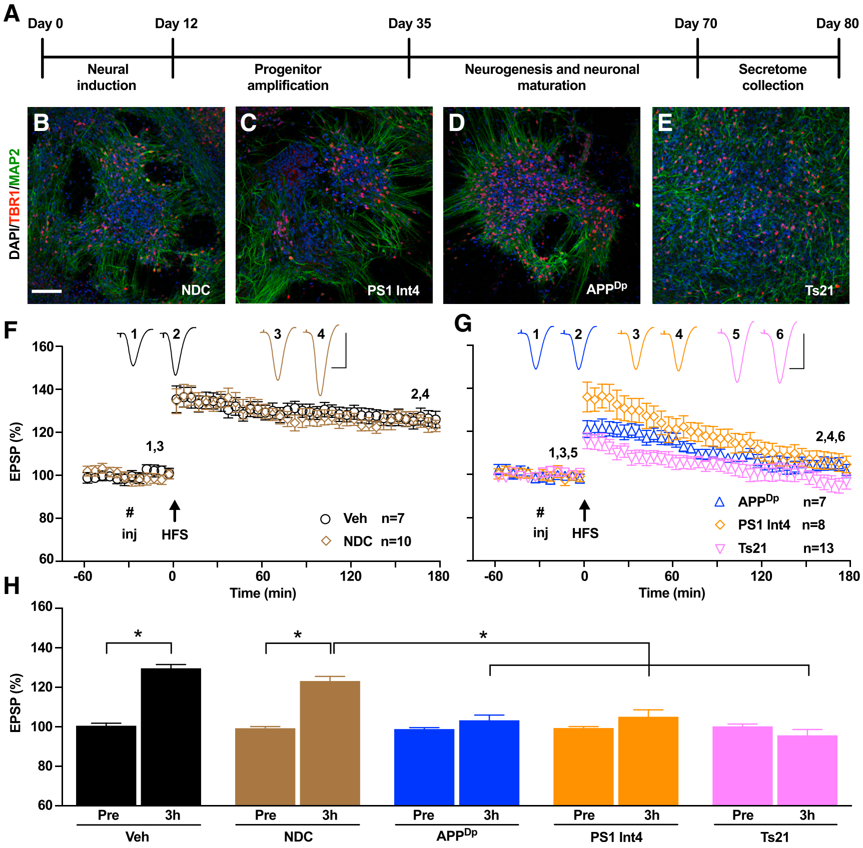

Figure 1. Inhibition of LTP *In Vivo* by Secretomes of Human Stem Cell Models of Alzheimer's Disease

(A) Scheme outlining the generation of cortical cultures and harvesting of secretomes from iPSCs.

(B–E) Representative confocal images confirming the differentiation of cortical neurons from NDC (B), PS1 Int4 (C), APP^{Dp} (D), and Ts21 (E) iPSCs of each genotype by the expression of TBR1 (red), a transcription factor expressed in layer 6 glutamatergic neurons, and neuron-specific MAP2 (green) in dendrites at day 80 post-neural induction. Scale bar, 100 μm.

(F) The application of high-frequency conditioning stimulation (HFS, arrow) in the hippocampal CA1 area of the anesthetized rat induced a robust and persistent LTP after an intracerebroventricular injection (# inj) of PBS vehicle or NDC secretome (mean ± SEM % pre-HFS baseline at 3 hr: Veh, 129.7 ± 1.8%; NDC, 123.2 ± 2.3%).

(G) Injection of secretomes from APP^{Dp} (103.3 ± 2.6%), PS1 Int4 (105.1 ± 3.4%), or Ts21 (95.7 ± 3.0%) neurons completely inhibited LTP at 3 hr post-HFS. The y axis is as shown in (F).

(H) Values represent the strength of synaptic transmission before (Pre) and 3 hr after the application of HFS for data in (F) and (G). $^{*}p < 0.05$, one-way ANOVA-Sidak and paired t test.

n, number of rats. Calibration bars for EPSP traces: vertical, 2 mV; horizontal, 10 ms.

See also Figures S1 and S4.

duplication (APP^{Dp}) (Israel et al., 2012), and Ts21 (Park et al., 2008)—were generated from iPSCs according to our previously described methods (Shi et al., 2012b). Secretomes were harvested at 48-hr intervals between days 70 and 80 post-neural induction from cultures of cortical glutamatergic neurons and astrocytes (Figures 1A–1E; Figures S1A–S1D). As we previously reported, APP^{Dp} and Ts21 cultures secreted more Aβ than NDC neurons (Figure S1E), and PS1 Int4 neurons exhibited altered Aβ40:Aβ42 and Aβ40:Aβ38 ratios (Figures S1F and S1G) (Moore et al., 2015). Cultures generated from each genotype were confirmed as being cortical in neuronal identity by immunostaining, gene expression, and western blotting, with the only notable difference in cellular composition being an increase in astrocyte number in the Ts21 cultures (Figure S1). Having validated the neuronal composition of the cultures and primary effects of each mutation on Aβ production, we investigated the effects of secretomes on synaptic LTP in anesthetized rats.

Synaptic plasticity was measured in the CA1 area of the dorsal hippocampus following injection of secretomes via a cannula into the lateral ventricle adjacent to the location of the implanted stimulating and recording electrodes under urethane anesthesia. The application of high-frequency conditioning stimulation (HFS) 30 min after the intracerebroventricular (i.c.v.) injection of NDC secretome (10 μL) triggered robust and stable LTP that was indistinguishable from LTP in vehicle-injected rats (Figures 1F and 1H). In contrast, injection of APP^{Dp}, PS1 Int4, and Ts21 secretomes all inhibited LTP (Figures 1G and 1H).

LTP Inhibition by PS1 Int4 and APP^{Dp} Secretomes Is Mediated by Aβ

The LTP-disrupting secretomes have altered levels or composition of Aβ peptides compared with that of NDC neurons (Figures S1 and S2). Therefore, we tested whether removal of Aβ could prevent LTP inhibition by each secretome. Immunodepletion (ID) of secretomes was performed with the pan-anti-Aβ-antiserum AW7, which recognizes multiple Aβ sequences and aggregation states but not APPsα or Aη peptides (Hong et al., 2018) (see Table S2 for a list of antibodies and their antigens/epitopes). As expected, AW7 ID effectively removed Aβ from neuronal secretomes so that neither Aβ40 nor Aβ42 was detected by ELISA (Figures 2A and 2B). LTP inhibition by APP^{Dp} and PS1 Int4 secretomes was prevented by AW7 ID, confirming that Aβ peptides are responsible for their synaptotoxic effects (Figures 2C and 2D). However, immunodepletion of Aβ peptides did not alter the LTP inhibition mediated by Ts21 secretome (Figures 2C and 2D).

We used size exclusion chromatography (SEC) to fractionate all the secretomes and assayed fractions using Aβ40 and Aβ42 ELISAs and western blotting (Figure S2). Both ELISAs detected a large Aβ monomer peak with negligible amounts of higher-molecular weight species. The APP^{Dp} secretome contained the highest levels of Aβ, and the PS1 Int4 secretome had the highest

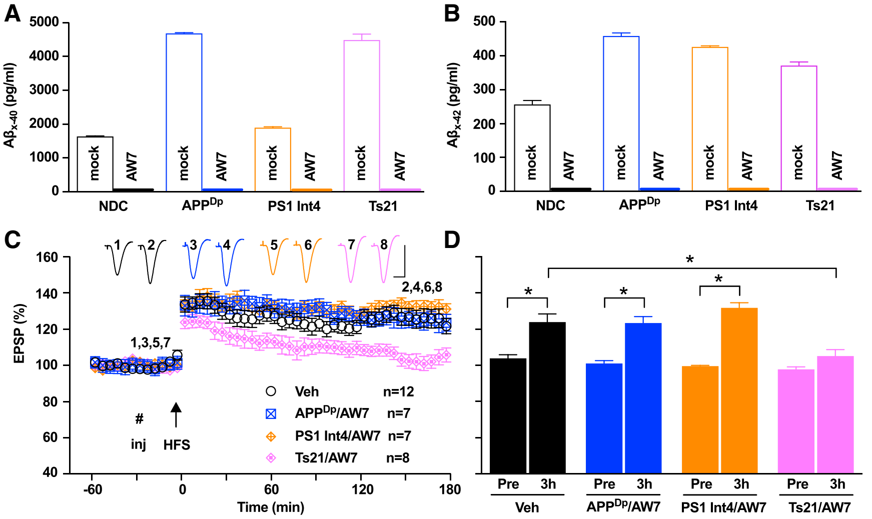

Figure 2. LTP Inhibition by PS1 Int4 or APP^{Dp} Secretomes Is Prevented by Immunodepletion with a Pan-Aβ Polyclonal Antibody

(A and B) Neuronal secretomes treated with pre-immune serum (Mock) contain Aβ40 (A) and Aβ42 (B) at levels readily detectable with immunoassays, whereas AW7 immunodepleted secretomes do not contain quantifiable levels of Aβ.

(C) Immunodepletion of Aβ peptides with AW7 rescued the inhibition of LTP by APP^{Dp} (APP^{Dp}/AW7: 123.4 ± 3.5%) and PS1 Int4 (PS1 Int4/AW7: 131.8 ± 2.8%), but not Ts21 (Ts21/AW7: 105.1 ± 3.7%). Calibration bars for EPSP traces: vertical, 2 mV; horizontal, 10 ms.

(D) Values before (Pre) and 3 hr post-HFS. The y axis is as shown in (C). *$p < 0.05$, one-way ANOVA-Sidak and paired t test.

See also Figures S2 and S4.

Aβ42/40 ratio. Otherwise, there were no notable differences between lines (Figures S2A and S2B). Western blotting with 6E10, a monoclonal antibody that recognizes Aβ, N-terminally extended (NTE)-Aβ, Aηα, and APPsα, also failed to reveal differences that could explain why PS1 Int4 and APP^{Dp} secretomes block LTP in an Aβ-dependent manner, whereas the block mediated by Ts21 secretome was independent of Aβ (Figure S2C).

Extracellular Tau Mediates the Blockade of LTP by the Secretome of Ts21 Neurons

Our functional analyses demonstrated that the plasticity-disrupting activity in APP^{Dp} and PS1 Int4 secretomes was mediated by a form of Aβ, whereas the activity in the secretome of Ts21 neurons was mediated by a distinct entity. Certain forms of tau are known to inhibit LTP (Fá et al., 2016; Lasagna-Reeves et al., 2012), and tau metabolism is altered in both fAD and Ts21 neurons (Hof et al., 1995; Mondragón-Rodríguez et al., 2014; Portelius et al., 2014). To address the potential involvement of tau, we immunodepleted the Ts21 secretome that previously had been immunodepleted of Aβ (Ts21/AW7), using a mid-region-directed anti-tau antibody, Tau5 (Figure S3A). This reduced the most abundant tau species in the Ts21 secretome by more than 80%, compared with an isotype control antibody (Figure 3A). Ts21 secretome mock-immunodepleted with the isotype control antibody impaired LTP, whereas immunodepletion of tau prevented the inhibition of LTP (Figures 3B and 3C).

To further examine whether synaptotoxic forms of tau were produced by each of the genetic models of AD studied here, an excess of Tau5 (2.5 μg) was co-injected with each secretome. Inhibition of LTP by Ts21/AW7 secretome was prevented by co-injection with Tau5, whereas the anti-Aβ antibody 6E10 (used as an isotype control) had no effect (Figures 3D and 3E). In contrast to results found with Ts21/AW7 secretome, co-injection of Tau5 did not prevent the inhibition of LTP by APP^{Dp} or PS1 Int4 secretomes (Figures 3F and 3G).

To compare the forms of extracellular tau released by neurons of each genotype, we analyzed their secretomes with four ELISAs, which, in combination, can differentiate between full-length (FL), mid-region (MR), N-terminal (NT), and C-terminal (CT) fragments of tau (Figure S3A; Table S3). The relative distribution of extracellular tau was comparable across lines, with MR-containing fragments the most prominent (Table S3). To further resolve different forms of tau, we size-fractionated secretomes from Ts21 and PS1 Int4, which showed that Ts21 secretome contained more SEC early-eluting MR- and CT-reactive material (Figures S3B–S3E). Although less sensitive than ELISA, western blotting detected a broad range of tau fragments and allowed resolution of different-sized species that elute in the same SEC fractions (Figures S3F and S3G). Tau fragments were more abundant in PS1 Int4 than Ts21 secretomes, with the exception of an ~11-kDa band that was at higher levels in the Ts21 secretome (Figures S3F and S3G, green boxes).

Independent iPSC Lines Recapitulate the Genotype-Specific Effects of PS1 Int4 and Ts21 Secretomes

We tested secretomes from neurons differentiated from independent non-diseased control (NDC.B), PS1 Int4 (PS1 Int4.B), and Ts21 (Ts21.B) iPSC lines (Figures S4A–S4D) to investigate the reproducibility of our findings. PS1 Int4.B iPSCs were derived from the same donor as in our initial experiments but were reprogrammed independently using a different method. Ts21.B iPSCs were generated from a second independent Ts21 donor. As before, secretomes from healthy control (NDC.B) neurons did not block LTP induction (Figures S4E and S4F). In contrast, PS1 Int4.B secretome blocked LTP, and this activity was relieved by ID of Aβ peptides with AW7 (Figures S4G–S4I), as was found with PS1 Int4 (Figure 2). As with Ts21 secretome (Figure 3), Ts21.B secretome blocked LTP, and this effect was prevented by tau immunodepletion (Figures S4J–S4L).

Synaptic Dysfunction Induced by PS1 Int4, APP^{Dp}, and Ts21 Secretomes Is Dependent on Cellular PrP

Given that PrP has been shown to be a mediator of Aβ-induced synaptic dysfunction (Laurén et al., 2009) and has been

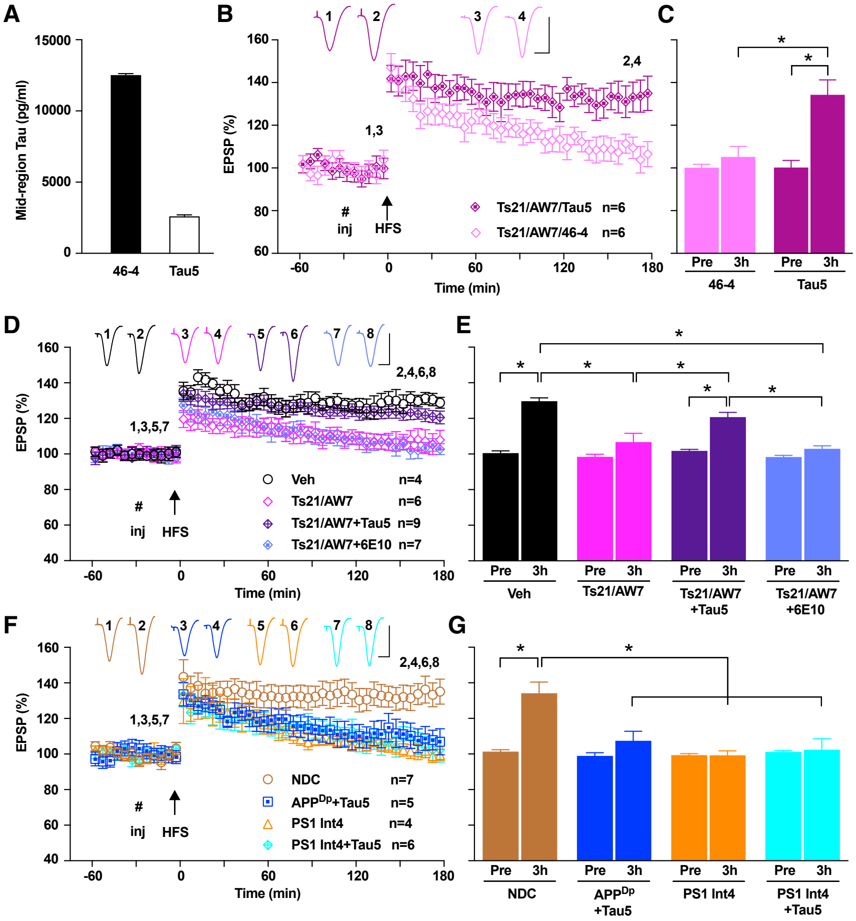

Figure 3. Extracellular Tau Mediates the Blockade of LTP by the Ts21 Secretome

(A) Immunodepletion of Ts21 secretome with Tau5 reduced the levels of mid-region containing tau to less than 20% of the original concentration. The monoclonal antibody 46-4 was used as an isotype control.

(B and C) Tau immunodepletion with Tau5 prevented the inhibition of LTP by the Ts21 secretome that had previously been immunodepleted with AW7, while mock ID with 46-4 did not (B). Data at 3 hr (Ts21/AW7/Tau5: 134.3 ± 7.0%; Ts21/AW7/46-4: 105.2 ± 4.9%) are summarized in (C). *$p < 0.05$, two-way ANOVA RM-Sidak and paired t test.

(D and E) Ts21 secretome that had previously been immunodepleted with AW7 inhibited LTP (Ts21/AW7: 106.7 ± 4.9%). Co-injection of Tau5 prevented inhibition of LTP (Ts21/AW7+Tau5: 120.8 ± 2.6%), while an isotype control antibody (6E10, 2.5 μg, i.c.v. injection) did not (Ts21/AW7+6E10: 102.9 ± 1.7%), as summarized in (D) and (E). The y axis is as shown in (D). *$p < 0.05$, one-way ANOVA-Sidak and paired t test.

(F and G) Stable LTP was induced by HFS 30 min after the i.c.v. injection of NDC secretome. Tau5 (2.5 μg, i.c.v. injection) was co-administered with APP^{Dp} secretome but did not affect the inhibition of LTP (APP^{Dp}+Tau5: 107.4 ± 5.4%). LTP was inhibited to a similar extent in animals treated with PS1 Int4 secretome (99.2 ± 2.5%) or co-treated with Tau5 (PS1 Int4+Tau5: 102.4 ± 6.2%), as summarized in (F) and (G). The y axis is as shown in (F). *$p < 0.05$, one-way ANOVA-Sidak and paired t test.

Calibration bars for EPSP traces: vertical, 2 mV; horizontal, 10 ms.

See also Figures S3 and S4.

suggested to act as a sensor for misfolded proteins (Resenberger et al., 2011), we investigated whether PrP was involved in the plasticity defects induced by neural secretomes. Pre-injection of 6D11, an antibody known to prevent Aβ binding to PrP (Laurén et al., 2009), prevented the disruptive effect of PS1 Int4 (Figures 4A and 4B) and APP^{Dp} (Figures 4C and 4D) secretomes on LTP. The inhibition of synaptic plasticity by Ts21 secretome was also prevented by 6D11 but not by an isotype control antibody (Figures 4E and 4F). Furthermore, 6D11 alone did not facilitate a control decremental LTP induced by a weak conditioning stimulation protocol (Figures 4G and 4H). Together, these data indicate that, although the synaptotoxic activity of PS1 Int4, APP^{Dp}, and Ts21 secretomes are mediated by distinct proteins, all require PrP to disrupt plasticity.

DISCUSSION

We report here the use of human iPSC-derived neuronal secretomes and the *in vivo* measurement of LTP to search for extracellular factors that impair synaptic plasticity. Impairment of LTP by APP^{Dp} and PS1 Int4 secretomes is mediated by soluble Aβ, whereas the active entity in Ts21 secretome is a soluble form of tau. The replication of these findings using independent PS1 Int4 and Ts21 secretomes supports the conclusion that several different synaptoxic proteins are released by human stem models of AD.

While the identity and, consequently, the concentration of the toxic species are currently unknown, we infer from our measurements of Aβ and tau that the concentration of active species is likely to be in the low nanomolar/picomolar range. Immediately obvious candidates for the active APP-derived synaptotoxic species in PS1 Int4 and APP^{Dp} secretomes include oligomeric forms of canonical Aβ (Lambert et al., 1998) or NTE-Aβ (Welzel et al., 2014), each of which have been shown to perturb LTP and are recognized by the AW7 antiserum. While we detected these and various other APP derivatives in the secretomes of all lines studied, we discerned no obvious qualitative difference that could explain why only the PS1 Int4 and APP^{Dp} secretomes block LTP in a manner prevented by immunodepletion of Aβ with the AW7 antibody.

Detailed analysis of secretomes using four distinct tau ELISAs, two different western blotting antibodies, and size fractionation revealed a complex mixture of extracellular tau species. Consistent with previous reports, we detected abundant mid-region fragments, lower levels of NT and CT fragments, and very low amounts of FL tau (Bright et al., 2015; Kanmert et al., 2015). While there were no obvious qualitative differences between the forms and levels of tau in Ts21 secretome compared with

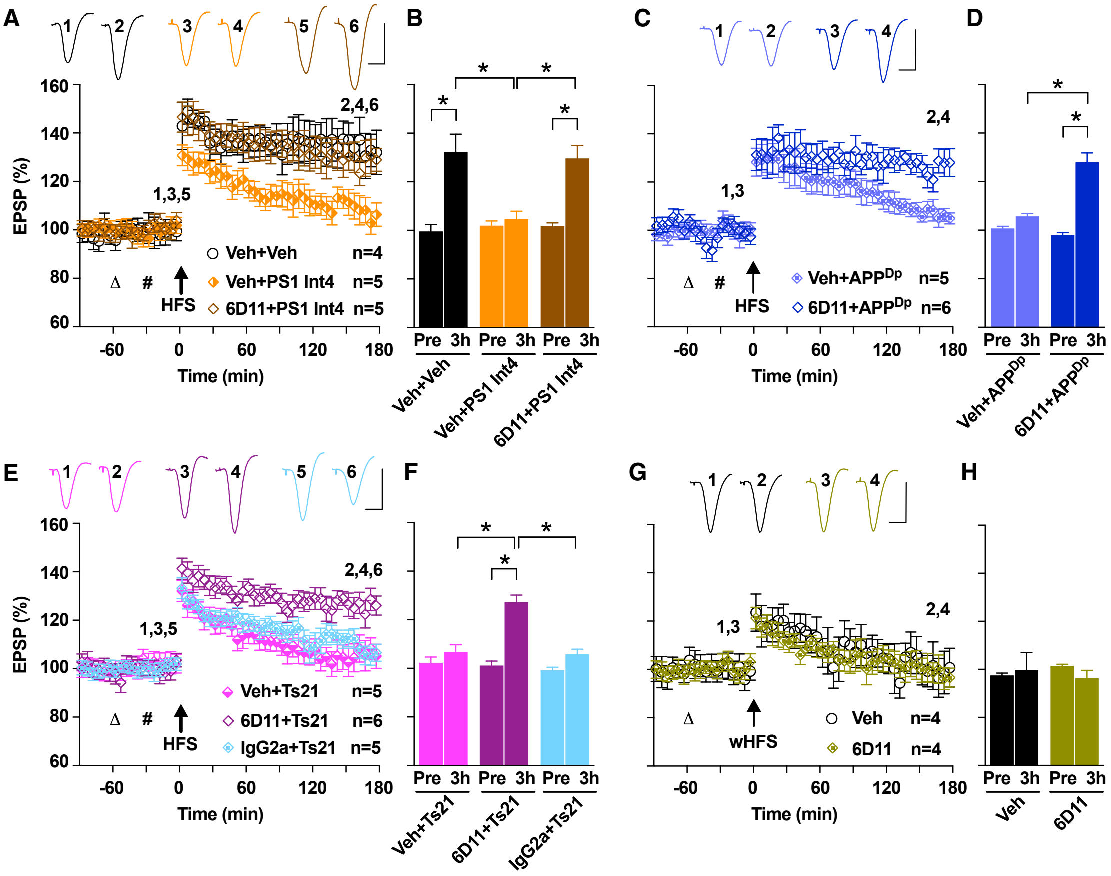

Figure 4. Synaptic Dysfunction Induced by PS1 Int4, APP^{Dp}, and Ts21 Secretomes Can Be Relieved by Targeting Cellular PrP

(A and B) The inhibition of LTP mediated by the PS1 Int4 secretome (104.6 ± 3.3%) was restored to the level of vehicle controls (132.4 ± 7.2%) by i.c.v. pre-injection (triangle) of 6D11 (6D11+PS1: 129.6 ± 5.3%) (A). Data at 3 hr post-HFS are summarized in (B). The y axis is as shown in (A). *$p < 0.05$, one-way ANOVA-Sidak and paired t test.

(C and D) Pre-injection of 6D11 also prevented the inhibition of LTP by the APP^{Dp} secretome (C). Data at 3 hr (Veh+APP^{Dp}: 105.7 ± 1.1%; 6D11+APP^{Dp}: 128.0 ± 3.8%) are summarized in (D). The y axis is as shown in (C). *$p < 0.05$, two-way ANOVA RM-Sidak and paired t test.

(E and F) The effect of Ts21 secretome (106.7 ± 3.1%) was blocked by 6D11 pre-injection (6D11+Ts21: 127.3 ± 2.9%), but not by an isotype control antibody (IgG2a+Ts21: 105.8 ± 2.1%), as summarized in (E) and (F). The y axis is as shown in (E). *$p < 0.05$, one-way ANOVA-Sidak and paired t test.

(G and H) A weak conditioning stimulation protocol (wHFS, arrow) induced a decremental LTP (Veh: 99.8 ± 7.2%), and 6D11 alone (96.4 ± 3.2%) did not facilitate this decremental LTP, as summarized in (G) and (H). The y axis is as shown in (G). *$p < 0.05$, two-way ANOVA RM-Sidak and paired t test.

Calibration bars for EPSP traces: vertical, 2 mV; horizontal, 10 ms.

the others studied here, we did detect two forms of tau that are increased in the Ts21 secretome compared with PS1 Int4. One is a low-abundance fragment that aberrantly elutes from SEC and lacks an intact N terminus, and the second is a more abundant ~11-kDa fragment that spans at least parts of the mid-region and microtubule-binding-region domains. As yet, it is unclear why a form of tau is the active species in secretomes from Ts21, but not PS1 and APP^{Dp} lines. Trisomy 21 has complex impacts on cellular biology due to widespread perturbation of the proteome (Liu et al., 2017), which may contribute to the differences in extracellular tau production found here.

It has previously been suggested that PrP might serve as a sensor for protein misfolding and that persistent binding of protein aggregates may have adverse effects (Resenberger et al., 2011). Here, we report that plasticity-disrupting activities mediated by extracellular forms of Aβ and tau can be prevented by antibody blockade of PrP. These results support further investigation of synaptotoxic proteins in the secretomes of iPSC-derived neurons and the PrP-dependent mechanism by which they exert their effects. Definitive identification of each synaptotoxic protein will enable further analysis of the cellular mechanisms underlying their ability to inhibit LTP.

EXPERIMENTAL PROCEDURES

Please see the Supplemental Experimental Procedures and Tables S1 and S2 for full details.

Study Approval

This research was carried out in accordance with the UK Code of Practice for the Use of Human Stem Cell Lines. The animal care and experimental protocols were carried out in accordance with the approval and oversight of the Irish Health Products Regulatory Authority, Dublin, Ireland.

Generation of Cortical Cultures and Secretome Collection

Pluripotent stem cells were cultured by standard methods and differentiated to cerebral cortex using established protocols (Shi et al., 2012b). Secretomes were collected at 48-hr intervals between days 70 and 80, after which the cultures were harvested for RNA and protein analysis or fixed for immunohistochemistry. Aβ38, Aβ40, and Aβ42 peptides were analyzed by multiplexed ELISA.

Secretome Processing and Immunoprecipitation

Secretomes were clarified by centrifugation and dialyzed against artificial cerebrospinal fluid (aCSF) before freezing at −80°C. Immunoprecipitation of Aβ was performed by 2 rounds of 12-hr incubation with AW7 (20 μL) and Protein A Sepharose beads at 4°C. Preimmune serum was used as a control. Tau5 (10 μg) and Protein G Agarose beads were used in 2 rounds of 12-hr incubations at 4°C to immunodeplete tau. The HIV coat protein 1 antibody, 46-4, was used as a control in tau immunoprecipitations.

SEC, ELISA, and Western Blot

Twelve-milliliter aliquots of ultracentrifuged and dialyzed secretomes were concentrated 10-fold (to 1.2 mL) using Amicon Ultra-15 3-kDa centrifugal filters (Millipore, Billerica, MA, USA) at 4°C. Immediately thereafter, 1 mL concentrate was chromatographed on tandem Superdex 200 Increase-Superdex 75 10/300 GL (GE Healthcare, Marlborough, MA, USA) columns eluted in 50 mM ammonium bicarbonate (pH 8.5) (Amersham Life Sciences, Uppsala, Sweden). One-milliliter fractions were collected and lyophilized for western blot or used for ELISA analysis.

Electrophysiology

Experiments were carried out on urethane-anesthetized (1.5–1.6 g/kg, intraperitoneally [i.p.]) male Lister Hooded rats (250–350 g), with the exception of 28 similarly sized Wistar rats that were used in the initial studies of the LTP disruptive effect of the Ts21 secretome. Hippocampal LTP was measured by recording field excitatory postsynaptic potentials (EPSPs) from the stratum radiatum of CA1 in response to stimulation of the ipsilateral Schaffer collateral/commissural pathway before and after 200-Hz HFS, as previously described (Hu et al., 2014). Secretomes were injected via cannula into the lateral ventricle of rats 30 min before the induction of synaptic plasticity. Tau5 and 6D11 (both 2.5 μg) were co-injected with secretomes, whereas 6D11 and immunoglobulin G2A (IgG2A) (both 20 μg) were injected 30 min before the application of secretomes to determine the requirement for tau and PrP binding, respectively.

Data Analysis

All statistical analyses of LTP were conducted in Prism v.6.07 (GraphPad Software, La Jolla, CA, USA). The magnitude of LTP is expressed as the percentage of pre-HFS baseline EPSP amplitude (±SEM). The n refers to the number of animals per group. Control experiments were interleaved randomly throughout. For timeline graphical representation, EPSP amplitudes were grouped into 5-min epochs; for statistical analysis, EPSP amplitudes were grouped into 10-min epochs. One-way ANOVA with Sidak's multiple comparison test (one-way ANOVA-Sidak) was used for comparisons between groups of three or more. Two-way ANOVA with repeated measures with Sidak's multiple comparison test (two-way ANOVA RM-Sidak) was used when there were only two groups. Paired t tests were carried out to compare pre- and post-HFS values within groups. A value of $p < 0.05$ was considered statistically significant.

SUPPLEMENTAL INFORMATION

Supplemental Information includes Supplemental Experimental Procedures, four figures, and three tables and can be found with this article online at https://doi.org/10.1016/j.celrep.2018.04.040.

ACKNOWLEDGMENTS

Research reported here was supported by Science Foundation Ireland (10/IN.1/B3001 and 14/IA/2571 to M.J.R.), a Wellcome Trust Investigator Award (WT101052MA to F.J.L.), a grant from the Alborada Trust and Alzheimer's Research UK Stem Cell Research Centre (ARUK-SCRC2017-1 to F.J.L.), and by the NIH (grants R01 AG046275 and R21 AG053827 to D.M.W.). We thank Dr. Zhicheng Chen for advice and assistance with certain tau assays. Research in F.J.L.'s group benefits from core support to the Gurdon Institute from the Wellcome Trust (ref. 203144) and Cancer Research UK (C6946/A24843). We are grateful to Ellie Tuck and Dr. Vickie Stubbs for technical support, Andy Billinton and Michael Perkinton (MedImmune) for providing the TauAβ antibody, and Dr. Frédérique Bard (Janssen Immunotherapy) for providing the anti-Aβ antibodies 266, 2G3, and 21F12.

AUTHOR CONTRIBUTIONS

M.J.R., D.M.W., and F.J.L. conceived the idea, directed experiments, and wrote the manuscript. S.M. generated and characterized iPSC-derived neurons and their secretomes. G.T.C. and T.T.O. clarified, dialyzed, immunodepleted, and characterized secretomes. N.-W.H. and I.K. conducted the electrophysiological experiments. All authors contributed to preparing figures and writing the manuscript.

DECLARATION OF INTERESTS

F.J.L. is the scientific founder of Gen2 Neuroscience. The other authors declare no competing interests.

Received: October 2, 2017
Revised: February 28, 2018
Accepted: April 6, 2018
Published: May 15, 2018

REFERENCES

Benilova, I., Karran, E., and De Strooper, B. (2012). The toxic Aβ oligomer and Alzheimer's disease: an emperor in need of clothes. Nat. Neurosci. *15*, 349–357.

Bright, J., Hussain, S., Dang, V., Wright, S., Cooper, B., Byun, T., Ramos, C., Singh, A., Parry, G., Stagliano, N., and Griswold-Prenner, I. (2015). Human secreted tau increases amyloid-beta production. Neurobiol. Aging *36*, 693–709.

Fá, M., Puzzo, D., Piacentini, R., Staniszewski, A., Zhang, H., Baltrons, M.A., Li Puma, D.D., Chatterjee, I., Li, J., Saeed, F., et al. (2016). Extracellular tau oligomers produce an immediate impairment of LTP and memory. Sci. Rep. *6*, 19393.

Goedert, M. (2016). The ordered assembly of tau is the gain-of-toxic function that causes human tauopathies. Alzheimers Dement. *12*, 1040–1050.

Goldstein, L.S., Reyna, S., and Woodruff, G. (2015). Probing the secrets of Alzheimer's disease using human-induced pluripotent stem cell technology. Neurotherapeutics *12*, 121–125.

Gómez-Ramos, A., Díaz-Hernández, M., Cuadros, R., Hernández, F., and Avila, J. (2006). Extracellular tau is toxic to neuronal cells. FEBS Lett. *580*, 4842–4850.

Hof, P.R., Bouras, C., Perl, D.P., Sparks, D.L., Mehta, N., and Morrison, J.H. (1995). Age-related distribution of neuropathologic changes in the cerebral cortex of patients with Down's syndrome. Quantitative regional analysis and comparison with Alzheimer's disease. Arch. Neurol. *52*, 379–391.

Hong, W., Wang, Z., Liu, W., O'Malley, T.T., Jin, M., Willem, M., Haass, C., Frosch, M.P., and Walsh D, M. (2018). Diffusible, highly bioactive oligomers represent a critical minority of soluble Aβ in Alzheimer's disease brain. Acta Neuropathol. https://doi.org/10.1007/s00401-018-1846-7.

Hu, N.W., Nicoll, A.J., Zhang, D., Mably, A.J., O'Malley, T., Purro, S.A., Terry, C., Collinge, J., Walsh, D.M., and Rowan, M.J. (2014). mGlu5 receptors and cellular prion protein mediate amyloid-β-facilitated synaptic long-term depression in vivo. Nat. Commun. *5*, 3374.

Hyman, B.T., Phelps, C.H., Beach, T.G., Bigio, E.H., Cairns, N.J., Carrillo, M.C., Dickson, D.W., Duyckaerts, C., Frosch, M.P., Masliah, E., et al. (2012).

National Institute on Aging-Alzheimer's Association guidelines for the neuropathologic assessment of Alzheimer's disease. Alzheimers Dement. *8*, 1–13.

Israel, M.A., Yuan, S.H., Bardy, C., Reyna, S.M., Mu, Y., Herrera, C., Hefferan, M.P., Van Gorp, S., Nazor, K.L., Boscolo, F.S., et al. (2012). Probing sporadic and familial Alzheimer's disease using induced pluripotent stem cells. Nature *482*, 216–220.

Kanmert, D., Cantlon, A., Muratore, C.R., Jin, M., O'Malley, T.T., Lee, G., Young-Pearse, T.L., Selkoe, D.J., and Walsh, D.M. (2015). C-terminally truncated forms of tau, but not full-length tau or its C-terminal fragments, are released from neurons independently of cell death. J. Neurosci. *35*, 10851–10865.

Lambert, M.P., Barlow, A.K., Chromy, B.A., Edwards, C., Freed, R., Liosatos, M., Morgan, T.E., Rozovsky, I., Trommer, B., Viola, K.L., et al. (1998). Diffusible, nonfibrillar ligands derived from Abeta1-42 are potent central nervous system neurotoxins. Proc. Natl. Acad. Sci. USA *95*, 6448–6453.

Lasagna-Reeves, C.A., Castillo-Carranza, D.L., Sengupta, U., Guerrero-Munoz, M.J., Kiritoshi, T., Neugebauer, V., Jackson, G.R., and Kayed, R. (2012). Alzheimer brain-derived tau oligomers propagate pathology from endogenous tau. Sci. Rep. *2*, 700.

Laurén, J., Gimbel, D.A., Nygaard, H.B., Gilbert, J.W., and Strittmatter, S.M. (2009). Cellular prion protein mediates impairment of synaptic plasticity by amyloid-beta oligomers. Nature *457*, 1128–1132.

Liu, Y., Borel, C., Li, L., Müller, T., Williams, E.G., Germain, P.-L., Buljan, M., Sajic, T., Boersema, P.J., Shao, W., et al. (2017). Systematic proteome and proteostasis profiling in human Trisomy 21 fibroblast cells. Nat. Commun. *8*, 1212.

Mondragón-Rodríguez, S., Perry, G., Luna-Muñoz, J., Acevedo-Aquino, M.C., and Williams, S. (2014). Phosphorylation of tau protein at sites Ser(396-404) is one of the earliest events in Alzheimer's disease and Down syndrome. Neuropathol. Appl. Neurobiol. *40*, 121–135.

Moore, S., Evans, L.D., Andersson, T., Portelius, E., Smith, J., Dias, T.B., Saurat, N., McGlade, A., Kirwan, P., Blennow, K., et al. (2015). APP metabolism regulates tau proteostasis in human cerebral cortex neurons. Cell Rep. *11*, 689–696.

Muratore, C.R., Rice, H.C., Srikanth, P., Callahan, D.G., Shin, T., Benjamin, L.N., Walsh, D.M., Selkoe, D.J., and Young-Pearse, T.L. (2014). The familial Alzheimer's disease APPV717I mutation alters APP processing and Tau expression in iPSC-derived neurons. Hum. Mol. Genet. *23*, 3523–3536.

Park, I.H., Arora, N., Huo, H., Maherali, N., Ahfeldt, T., Shimamura, A., Lensch, M.W., Cowan, C., Hochedlinger, K., and Daley, G.Q. (2008). Disease-specific induced pluripotent stem cells. Cell *134*, 877–886.

Portelius, E., Soininen, H., Andreasson, U., Zetterberg, H., Persson, R., Karlsson, G., Blennow, K., Herukka, S.K., and Mattsson, N. (2014). Exploring Alzheimer molecular pathology in Down's syndrome cerebrospinal fluid. Neurodegener. Dis. *14*, 98–106.

Resenberger, U.K., Harmeier, A., Woerner, A.C., Goodman, J.L., Müller, V., Krishnan, R., Vabulas, R.M., Kretzschmar, H.A., Lindquist, S., Hartl, F.U., et al. (2011). The cellular prion protein mediates neurotoxic signalling of β-sheet-rich conformers independent of prion replication. EMBO J. *30*, 2057–2070.

Shi, Y., Kirwan, P., Smith, J., MacLean, G., Orkin, S.H., and Livesey, F.J. (2012a). A human stem cell model of early Alzheimer's disease pathology in Down syndrome. Sci. Transl. Med. *4*, 124ra29.

Shi, Y., Kirwan, P., Smith, J., Robinson, H.P., and Livesey, F.J. (2012b). Human cerebral cortex development from pluripotent stem cells to functional excitatory synapses. Nat. Neurosci. *15*, 477–486, S1.

Spires-Jones, T.L., and Hyman, B.T. (2014). The intersection of amyloid beta and tau at synapses in Alzheimer's disease. Neuron *82*, 756–771.

Sweatt, J.D. (2016). Neural plasticity and behavior – sixty years of conceptual advances. J. Neurochem. *139* (*Suppl 2*), 179–199.

Walsh, D.M., Hartley, D.M., and Selkoe, D. (2003). The many faces of Aß: structures and activity. Curr. Med. Chem. - Immunol. Endocr. Metab. Agents *3*, 277–291.

Welzel, A.T., Maggio, J.E., Shankar, G.M., Walker, D.E., Ostaszewski, B.L., Li, S., Klyubin, I., Rowan, M.J., Seubert, P., Walsh, D.M., and Selkoe, D.J. (2014). Secreted amyloid β-proteins in a cell culture model include N-terminally extended peptides that impair synaptic plasticity. Biochemistry *53*, 3908–3921.

Woodruff, G., Young, J.E., Martinez, F.J., Buen, F., Gore, A., Kinaga, J., Li, Z., Yuan, S.H., Zhang, K., and Goldstein, L.S. (2013). The presenilin-1 ΔE9 mutation results in reduced γ-secretase activity, but not total loss of PS1 function, in isogenic human stem cells. Cell Rep. *5*, 974–985.

Yagi, T., Ito, D., Okada, Y., Akamatsu, W., Nihei, Y., Yoshizaki, T., Yamanaka, S., Okano, H., and Suzuki, N. (2011). Modeling familial Alzheimer's disease with induced pluripotent stem cells. Hum. Mol. Genet. *20*, 4530–4539.

Yang, T., Li, S., Xu, H., Walsh, D.M., and Selkoe, D.J. (2017). Large soluble oligomers of amyloid β-protein from Alzheimer brain are far less neuroactive than the smaller oligomers to which they dissociate. J. Neurosci. *37*, 152–163.

Cell Reports, Volume *23*

Supplemental Information

Extracellular Forms of Aβ and Tau

from iPSC Models of Alzheimer's Disease

Disrupt Synaptic Plasticity

Neng-Wei Hu, Grant T. Corbett, Steven Moore, Igor Klyubin, Tiernan T. O'Malley, Dominic M. Walsh, Frederick J. Livesey, and Michael J. Rowan

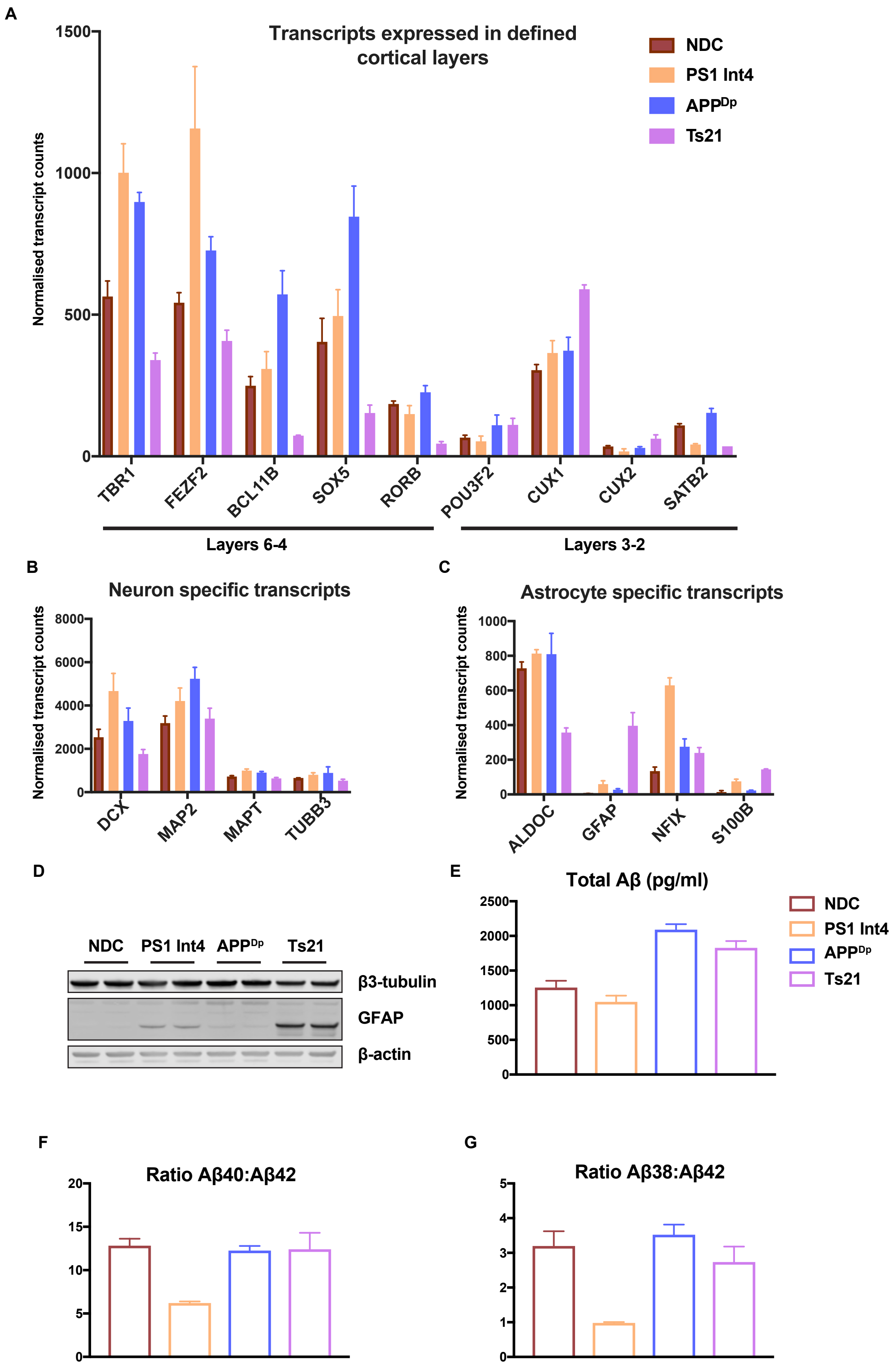
A
Transcripts expressed in defined cortical layers
Normalised transcript counts
NDC
PS1 Int4
APPDp
Ts21
TBR1
FEZF2
BCL11B
SOX5
RORB
POU3F2
CUX1
CUX2
SATB2
Layers 6-4
Layers 3-2
B
Neuron specific transcripts
DCX
MAP2
MAPT
TUBB3
C
Astrocyte specific transcripts
ALDOC
GFAP
NFIX
S100B
D
NDC
PS1 Int4
APPDp
Ts21
β3-tubulin
GFAP
β-actin
E
Total Aβ (pg/ml)
F
Ratio Aβ40:Aβ42
G
Ratio Aβ38:Aβ42

Figure S1. Generation of iPSC-derived human neuronal cultures; Related to Figure 1.

(A) Nanostring analysis of RNA extracted at day 80 post induction. Transcripts associated with deep and upper layer cortical neuronal subtypes were observed in cells derived from each genotype.
(B-C) Transcripts associated with both neuronal and astrocyte identities were expressed by cultures of each genotype, reflecting the composition of the cultures. The data presented in A-C were generated from 3 technical replicates and normalised to the expression levels of a set of 11 housekeeping genes; please see Supplemental Experimental Procedures for more detail. Error bars = S.D.
(D) Western blots of protein extracted at day 80 post induction. PS1 Int4 and APP^{Dp} cultures expressed similar levels of β3-tubulin to NDC. Ts21 cultures exhibited marginally lower expression of β3-tubulin than NDC but had increased expression of the astrocyte marker GFAP, consistent with the transcriptional analysis presented in (B-C). β-actin was used as a loading control for protein levels.
(E) APP^{Dp} and Ts21 neuronal cultures released higher concentrations of total Aβ peptides (Aβ38, Aβ40 and Aβ42) in accordance with their increased APP dosage.
(F-G) PS1 Int4 secretome exhibited a relative increase in Aβ42 compared with Aβ40 (F) and Aβ38 (G), due to the reduction in γ-secretase processivity that we previously described (Moore et al., 2015). The data presented in E-F were generated from 3 technical replicates. Error bars = S.D.

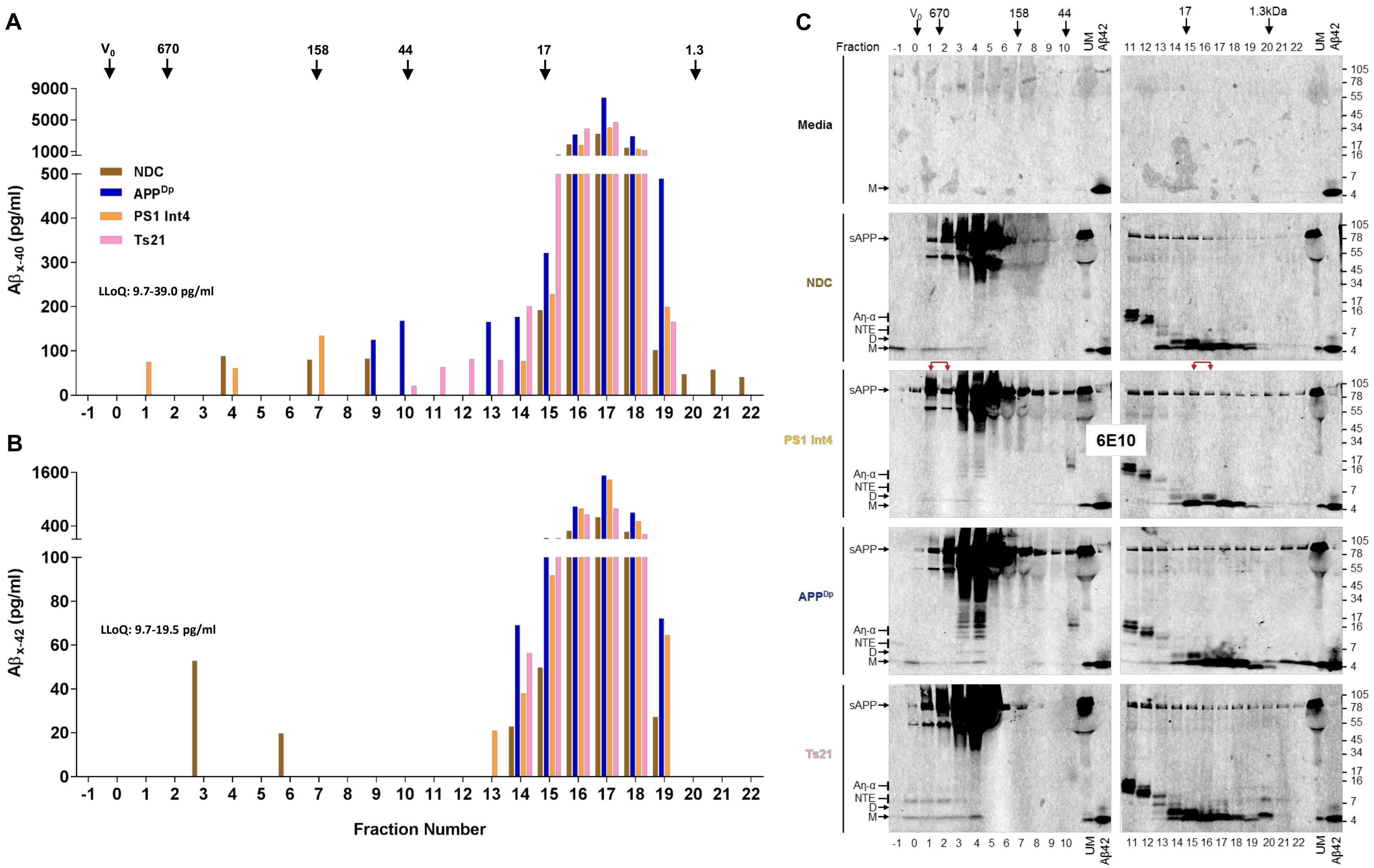
A
B
C
V0
670
158
44
17
1.3
NDC
APPDp
PS1 Int4
Ts21
LLoQ: 9.7-39.0 pg/ml
Aβx-40 (pg/ml)
LLoQ: 9.7-19.5 pg/ml
Aβx-42 (pg/ml)
Fraction Number
Fraction
1.3kDa
UM
Aβ42
Media
sAPP
Aη-α
NTE
D
M
6E10

Figure S2. Secretomes contain an array of APP fragments, including Aβ monomers and dimers, N-terminally extended Aβ and Aη-α; Related to Figure 2.

(A-C) Secretomes from non-demented control (NDC; brown), APP^{Dp} (blue), PS1 Int4 (orange), and Ts21 (pink) neurons were concentrated ten-fold and 1 mL of each concentrate used for SEC. Aliquots of SEC fractions were analyzed for Aβx-40 (A) and Aβx-42 (B) using MSD-based immunoassays. Fraction numbers are indicated on the x-axis and Aβ concentration on the y-axis, and the lower limit of quantitation (LLoQ) of each assay is provided. The elution of globular protein standards (the molecular weight of which are given in kDa) is indicated by downward pointing arrows on the top chromatogram and the void volume, determined from the elution of Blue dextran, is denoted as V_0.

The remainder (0.75 mL) of fractions used in A and B were lyophilized and then analyzed by western blotting. The monoclonal antibody, 6E10, recognizes residues 702-706 of APP_{770}, and detects multiple APP metabolites, including APPsα, Aη-α, N-terminally extended (NTE)-Aβ and Aβ and was used for all blots (C). V_o and elution of globular standards is indicated on the top blot, and SEC fraction numbers are indicated on the top and bottom blots. The secretomes analyzed are indicated on the left. Plain media (Media) was chromatographed, fractions lyophilized and used for western blotting to assess non-specific staining by primary or secondary antibodies. Migration of SDS-PAGE molecular weight standards (in kDa) is indicated on the right of each blot. Arrows indicate Aβ monomer (M), Aβ dimer (D) and sAPP, while dashes indicate NTE-Aβ and Aη-α species. Secretomes that had been concentrated but not chromatographed (designated UM) were mixed with sample buffer and loaded without further manipulation (10 mL). Synthetic Aβ (5 ng) was loaded in the last lane of each blot.

Red arrows above the PS1 Int4 blots in panel C indicate pairs of fractions that were loaded in the reverse order, resulting in exchange of the positions of fractions 1 and 2 on the left blot and fractions 15 and 16 on the right blot.

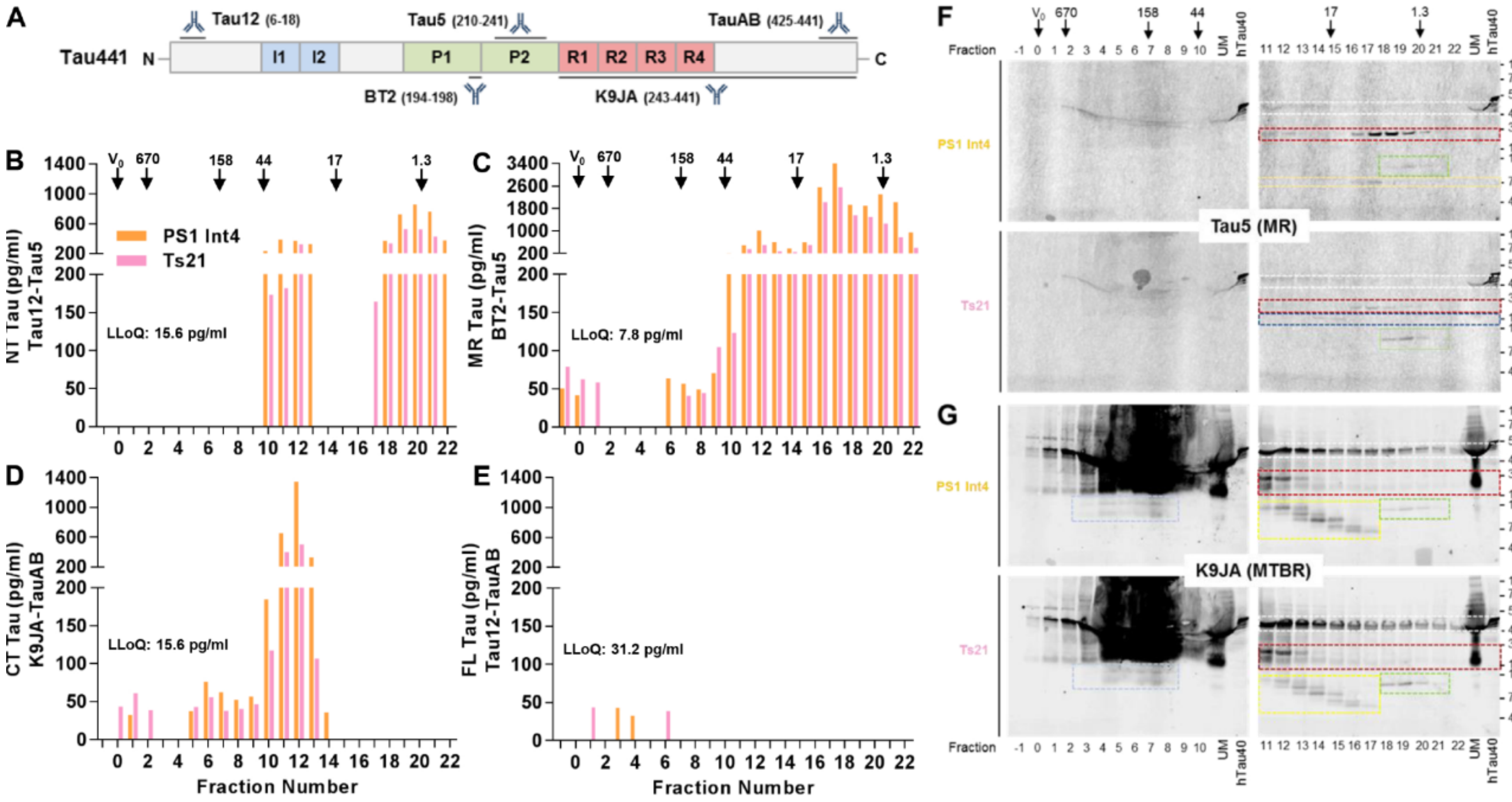

A
Tau441
N
C
Tau12 (6-18)
Tau5 (210-241)
TauAB (425-441)
BT2 (194-198)
K9JA (243-441)
I1
I2
P1
P2
R1
R2
R3
R4
B
NT Tau (pg/ml)
Tau12-Tau5
PS1 Int4
Ts21
LLoQ: 15.6 pg/ml
C
MR Tau (pg/ml)
BT2-Tau5
LLoQ: 7.8 pg/ml
D
CT Tau (pg/ml)
K9JA-TauAB
LLoQ: 15.6 pg/ml
E
FL Tau (pg/ml)
Tau12-TauAB
LLoQ: 31.2 pg/ml
Fraction Number
F
G
Fraction
Tau5 (MR)
K9JA (MTBR)
UM
hTau40

Figure S3. Secretomes contain a broad range of truncated tau species, the most abundant of which are mid-region fragments; Related to Figure 3.

(A) Schematic depicting the longest splice isoform of human tau which includes two N-terminal inserts (blue) and four C-terminal repeats (red). Epitopes of the antibodies used for ELISA and western blot are indicated by dark grey lines.

(B-E) Secretomes from PS1 Int4 (orange) and Ts21 (pink) iPSC-derived cortical neurons were concentrated ten-fold and 1 mL of each concentrate used for SEC. Aliquots of SEC fractions were analyzed for tau using 4 different ELISAs. These include N-terminal (NT; B), mid-region (MR; C), C-terminal (CT; D) and full-length (FL; E) assays. The capture and detection antibodies are listed on the y-axes. Fraction numbers are indicated on the x-axis and tau concentration on the y-axis, and the lower limit of quantitation (LLoQ) of each assay is provided. The elution of globular protein standards (the molecular weight of which are given in kDa) is indicated by downward pointing arrows on the top chromatogram and the void volume, determined from the elution of Blue dextran, is denoted as V_0.

The remainder (0.75 mL) of the fractions used in B-E were lyophilized and then analyzed by western blotting using both the mouse antibody, Tau5 (F) and the rabbit antiserum, K9JA (G). The signals for Tau5 and K9JA were detected simultaneously using the two-channel LiCor infrared imaging system and appropriate IR-labeled secondary antibodies. In both F and G, secretomes from PS1 Int4 are shown in the upper panels and Ts21 in lower panels. V_o and elution of globular standards is indicated on the top blot, and SEC fraction numbers are indicated on the top and bottom blots. Migration of SDS-PAGE molecular weight standards (in kDa) is indicated on the right of each blot and colored boxes are used to highlight the position of certain tau species. Unfractionated media (UM) and recombinant human tau 441 (10 ng) were loaded in the last two lanes of each gel.

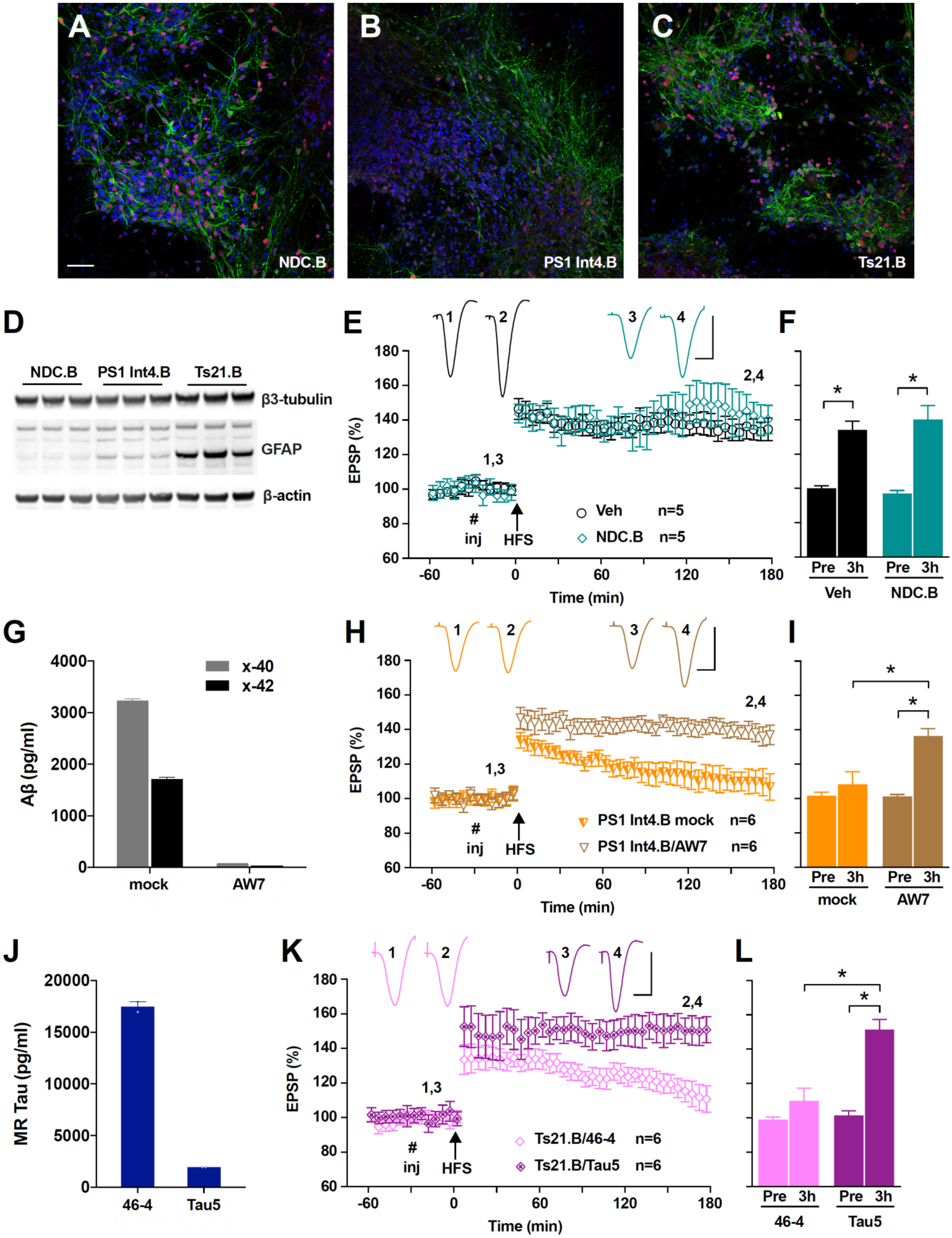
A
NDC.B
B
PS1 Int4.B
C
Ts21.B
D
NDC.B
PS1 Int4.B
Ts21.B
β3-tubulin
GFAP
β-actin
E
EPSP (%)
Time (min)
1,3
2,4
inj
HFS
Veh n=5
NDC.B n=5
F
Pre 3h
Veh
NDC.B
G
Aβ (pg/ml)
x-40
x-42
mock
AW7
H
EPSP (%)
Time (min)
PS1 Int4.B mock n=6
PS1 Int4.B/AW7 n=6
I
mock
AW7
J
MR Tau (pg/ml)
46-4
Tau5
K
EPSP (%)
Time (min)
Ts21.B/46-4 n=6
Ts21.B/Tau5 n=6
L
46-4
Tau5

Figure S4. Independent iPSC lines recapitulate the genotype-specific effects of PS1 Int4 and Ts21 secretomes; Related to Figures 1, 2 and 3.

(A-C) Representative confocal images confirming the induction of cortical neurons by the expression of TBR1 (red) and MAP2 (green) in neurons of each genotype at day 80 post neural induction. Scale bar: 100 μm.
(D) Western blots of protein extracted at day 80 post-induction. PS1 Int4.B and Ts21.B cultures expressed comparable levels of β3-tubulin to NDC.B controls. GFAP expression was increased in Ts21.B cultures, consistent with the analysis of Ts21 cultures presented in Figure S1.
(E, F) Intracerebroventricular injection (inj, #) of NDC.B secretome 30 min prior to the application of HFS did not affect LTP, with a magnitude (140.1 ± 8.2% at 3 h) comparable to vehicle-injected controls (134.0 ± 5.0%). Data at 3 h post-HFS are summarized in (F).
(G) Immunodepletion of PS1 Int4.B secretome with the pan-Aβ-antiserum AW7 reduced the levels of Aβ40 and Aβ42 to below the limit of detection by ELISA compared with the same secretome treated with control pre-immune serum (Mock).
(H, I) Injection of secretome from PS1 Int4.B treated with control pre-immune serum (mock) completely inhibited LTP at 3 h post-HFS (PS1 Int4.B: 108.2 ± 7.4%). Immunodepletion of Aβ with AW7 prevented the inhibition of LTP by PS1 Int4.B (PS1 Int4.B/AW7: 136.5 ± 4.1%). Data at 3 h post-HFS are summarized in (I).
(J) ID of Ts21.B secretome with the monoclonal antibody Tau5 reduced the levels of mid-region containing tau to less than 12% of the original concentration. The monoclonal antibody 46-4 was used as an isotype control.
(K, L) Immunodepletion of tau with Tau5 ID prevented the inhibition of LTP by the Ts21.B secretome (Ts21.B /Tau5: 151.3 ± 6.1%), while mock ID with 46-4 did not (Ts21.B /46-4: 109.8 ± 7.5%). Data at 3 h post-HFS are summarized in (L).
* $P<0.05$, two-way ANOVA RM-Sidak and paired *t*. Calibration bars for EPSP traces: vertical, 2 mV; horizontal, 10 ms.

SUPPLEMENTAL EXPERIMENTAL PROCEDURES

Generation of cortical cultures and secretome collection

The iPSC lines used in the this study were NDC (Israel et al., 2012), PS1 Int4 (Moore et al., 2015), APP^{Dp} (Israel et al., 2012) and Ts21 (Park et al., 2008). AD2-1 (here named NDC.B) and SFC808 (here named PS1 Int4.B) iPSCs were obtained from the StemBANCC consortium. Ts21.B iPSCs were generated from a fibroblast biopsy of an individual with Down syndrome using the CytoTune-iPS 2.0 Sendai Reprogramming Kit (ThermoFisher). Human pluripotent stem cells were maintained on Geltrex in Essential 8 media (both ThermoFisher). With minor modifications to account for feeder-free maintenance, directed differentiation of iPSCs to cerebral cortex was performed as previously described (Saurat et al., 2016; Shi et al., 2012a; Shi et al., 2012c). Briefly, multiple iPSC cultures per genotype were induced to form neuroepithelial sheets over the course of 12 days. Cortical progenitors were enriched and expanded between day 12 and 30 before the cultures were dissociated, mixed, aliquoted and frozen in liquid nitrogen. Cortical differentiations were performed by thawing progenitors from each genotype and passaging them into over 30 technical replicates at day 35 post induction. Secretomes of differentiated cultures was collected between day 70 and day 80 post-neural induction at 48 h intervals, aliquoted and stored in protein lo-bind tubes at -80°C.

Immunocytochemistry and imaging

Cultures were fixed at day 80 post neural induction in 4% paraformaldehyde in phosphate buffered saline before immunostaining with TBR1 and MAP2. Nuclei were stained by the addition of DAPI. Imaging was performed on an Olympus FV1000 inverted confocal microscope and processed with Fiji.

Nanostring profiling of cortical cultures

Cultures were harvested at day 80 post neural induction and RNA was extracted using TRIzol (ThermoFisher) before a custom probe set was used to assay transcriptional profile. Hybridization reactions were performed with 50 ng of RNA before post-hybridization and data collection was performed with the nCounter SPRINT Profiler (NanoString Technologies). The data were processed using the nSolver Analysis Software by subtracting the background of the geometric mean of 8 negative controls before normalization to the geometric means of 6 positive

controls and 11 housekeeping genes (CLTC, GAPDH, GUSB, HPRT1, PGK1, PPIA, RPLP1, RPS15A, RPS9, TBP, UXT). This assay was performed on technical triplicates of cultures from each genotype.

Western blot profiling of cortical cultures

Cultures were lysed in RIPA buffer (Sigma) supplemented with cOmplete protease inhibitors and PhosSTOP phosphatase inhibitors (both Roche) at day 80 post neural induction. Western blots were carried out using antibodies to β3-tubulin, GFAP and β-actin and imaged using the LI-COR Odyssey CLx Infrared Imaging System and Image Studio software.

Multiplexed Aβ ELISA

Quantification of Aβ38, Aβ40 and Aβ42 was carried out with multiplexed Meso Scale Discovery assay kits (K15200E) on a Quickplex SQ120 instrument (Meso Scale Discovery, Maryland) using 25μl of cell culture supernatant collected at day 78 post induction.

Clarification and dialysis of secretomes

Secretome was thawed, pooled into 50 mL batches and cleared of cell debris and extracellular vesicles by centrifugation. First, secretome was centrifuged at 200 × *g* and 4°C for 10 min. Then, the upper 97% was recovered (supernatant 1; S1) and centrifuged at 2,000 × *g* and 4°C for 10 min. Next, the upper 97% of this was recovered (S2) and centrifuged at 10,000 × *g* and 4°C for 30 min. Finally, the upper 97% was recovered (S3) and centrifuged at 100,000 × *g* and 4°C for 70 min. The upper 97% of the final supernatant (~44 mL) was dialyzed (using Slide-A-Lyzer™ G2 Dialysis Cassettes, 2K MWCO, ThermoFisher) against artificial cerebrospinal fluid (aCSF; 124 mM NaCl, 2.8 mM KCl, 1.25 mM NaH_2PO_4, 26 mM $NaHCO_3$) to remove bioactive small molecules. Dialysis was performed at 4°C against a 100-fold excess of aCSF with buffer changed three times over a 48 h period. Dialyzed secretome was divided into 1 mL aliquots and either frozen at -80°C or used immediately for immunoprecipitation.

Immunodepletion

Secretome (1 mL) was treated in one of two ways: (i) Immunodepleted (ID) of Aβ by 2 rounds of 12 h incubations with the purified anti-Aβ antiserum, AW7 (20 μL) and Protein A Sepharose beads (PAS; 10 μL; ThermoFisher) at 4°C. (ii) Parallel secretome samples were 'mock' ID with purified preimmune serum (PIS; 20 μL) and PAS (10 μL). In each case, samples were cleared of

beads and then both the ID and 'mock' ID samples were incubated with PAS alone to remove previously unbound IgG. To deplete tau species from secretome that had previously been ID with AW7 or PIS, 1 mL aliquots were incubated with the anti-tau monoclonal antibody (mAb) Tau5 (10 µg) and Protein G Agarose beads (PAG) beads (10 µL; Roche) for 2 rounds of 12 h incubations at 4°C. Parallel AW7 or PIS-ID secretome samples were 'mock' ID with 10 µg of the anti-HIV coat protein 1 mAb, 46-4, and PAG (10 µL). Both the Tau5 and 46-4 treated samples were cleared of beads and then both the ID and 'mock' ID samples were incubated with PAG alone to remove previously unbound IgG. ID supernatants were divided into 20 µL aliquots and stored at -80°C until analysis.

Aβx-40/x-42 immunoassays

Analysis of secretomes following clarification, dialysis and immunodepletion utilized highly sensitive, in-house Meso Scale Discovery (MSD) platform-based immunoassays. Unless otherwise indicated, reagents were from Meso Scale (Rockville, MD) and assays were conducted essentially as described previously (Mably et al., 2015). Multi-Array® 96-well small-spot black microplates were coated with 3 µg/mL of monoclonal antibody 266 (Table S2) in tris-buffered saline (TBS) and incubated at room temperature (RT) for 18 h. Unoccupied binding sites were blocked in 150 µL 5% Blocker A in TBS containing 0.05% Tween 20 (TBST) and agitated at 400 rpm for 1 h at 22°C. Plates were then washed three times with 150 µL TBST before samples (diluted 1:1 with 1% Blocker A in TBST) and standards were applied in triplicate and agitated at 400 rpm for 2 h at RT. After capture, plates were washed three times with 150 µL TBST and incubated with biotinylated 2G3 and 21F12 antibodies for x-40 and x-42 detection, respectively. Simultaneously, 1 µg/mL of the reporter reagent (SULFO-TAG labeled streptavidin) was added to diluent and incubated at RT with gentle agitation for 2 h. Finally, plates were washed three times with 150 µL TBST before 2 × MSD read buffer (150 µl per well) was applied to allow for electrochemiluminescence detection. A Sector imager was used to measure the intensity of emitted light, thus allowing the quantitative measurement of analytes present in the samples. The LLoQ, determined by calculating the average + 9 standard errors and 100 ± 20% recovery for each standard, was 39.06 pg/mL and 9.76 pg/mL for x-40 and x-42 assays, respectively.

Tau enzyme-linked immunosorbent assays (ELISAs)

Assays for mid-region tau, N-terminal (NT), C-terminal tau (CT1 and CT2) and full-length (FL) tau were performed using the same procedure but with different combinations of capture and detection antibodies (Kanmert et al., 2015). The antibodies employed, their epitopes, the tau fragment(s) recognized and LLoQs are described in (Table S2). For all assays, the capture antibody was coated at 2.5 µg/mL in TBS for 1 h at 37°C and 300 rpm. Plates were then washed three times with 100 µL TBST prior to blocking in 100 µL TBS containing 3% BSA for 2 h at RT and 300 rpm. Plates were washed three times with 100 µL TBST before 25 µL samples (diluted 1:1 in TBS containing 1% BSA) and standards were applied in triplicate and agitated for 16 h at 4°C. Importantly, the same calibration standard (recombinant human tau441) was used for all assays, thus enabling comparison of concentrations detected by different assays. The following day, 25 µL alkaline phosphatase conjugated detection antibodies diluted 1:250 in TBST containing 1% BSA were added directly to the plates without washing and incubated for 1 h at RT and 300 rpm. Finally, plates were washed three times with 100 µL TBST before 50 µL Tropix Sapphire II (Applied Biosystems) detection reagent was added and incubated for 30 min at RT and 300 rpm. Standard curves were fitted to a five-parameter logistic function with $1/Y^2$ weighting using MasterPlex ReaderFit (MiraiBio). LLoQs were calculated as described for the MSD assays and are provided in Fig. 4.

Animals and surgery

Experiments were carried out on urethane-anesthetized male Lister Hooded rats (250-350 g), with the exception of 28 similarly sized Wistar rats that were used in the initial studies of the LTP disruptive effect of the Ts21 secretome. Since Ts21 secretome had the same inhibitory effect in both strains (28 Wistar and 65 Lister Hooded rats) we combined these data. The animals were housed under a 12h light-dark cycle at room temperature (19-22°C). Prior to the surgery, animals were anesthetized with urethane (1.5-1.6 g/kg, i.p.). Lignocaine (10 mg, 1% adrenaline, s.c.) was injected over the area of the skull where electrodes and screws were to be implanted. The body temperature of the rats was maintained at 37-38 °C with a feedback-controlled heating blanket. A stainless-steel cannula (22 gauge, 0.7 mm outer diameter) was implanted above the right lateral ventricle (1mm lateral to the midline and 4 mm below the surface of the dura) for injecting antibody or the secretome. Intracerebroventricular (i.c.v.) injection was made via an internal cannula (28 gauge, 0.36 mm outer diameter). The solutions were injected in a volume up to 20 µL with a 1.5 µL/min speed. The vehicle injection contained phosphate buffered saline. Because there were no differences between the effects of naïve and

preimmune serum 'mock' immunodepleted samples, we combined these results. We carried out initial unblind studies followed by blind studies interleaved with unblind vehicle experiments. For analysis we combined these results with the exception of the data presented for the replication lines in Fig. S4, where we only included blind studies. Verification of the placement of cannula was performed postmortem by checking the spread of ink dye after i.c.v. injection.

Electrophysiology

Electrodes were made and implanted as described previously (Hu et al., 2014). Briefly, twisted bipolar stimulating electrodes were constructed from Teflon-coated tungsten wires (50 µm inner core diameter, 75 µm external diameter) and monopolar recording electrodes were constructed from Teflon-coated tungsten wires (75 µm inner core diameter, 112 µm external diameter) separately. Field excitatory postsynaptic potentials (EPSPs) were recorded from the stratum radiatum in the CA1 area of the right hippocampus in response to stimulation of the ipsilateral Schaffer collateral-commissural pathway. Electrode implantation sites were identified using stereotaxic coordinates relative to bregma, with the recording site located 3.4 mm posterior to bregma and 2.5 mm lateral to midline, and stimulating site 4.2 mm posterior to bregma and 3.8 mm lateral to midline. The final placement of electrodes was optimized by using electrophysiological criteria and confirmed via postmortem analysis.

Test EPSPs were evoked by square wave pulses (0.2 ms duration) at a frequency of 0.033 Hz and an intensity that triggered a 50% maximum response. LTP was induced using 200 Hz high frequency stimulation (HFS) consisting of one set of ten trains of twenty pulses (inter-train interval of 2 s). The stimulation intensity was raised to trigger EPSPs of 75% maximum during the HFS. The conditioning stimulation protocol did not elicit any detectible abnormal changes in background EEG, which was recorded from the hippocampus throughout the experiments. In some animals, a weak high frequency stimulation (wHFS, consisting of 10 trains of 10 pulses at 200 Hz with inter-train interval of 2 s) was used to induce decremental LTP.

Size exclusion chromatography and western blot analysis

Twelve milliliter aliquots of ultracentrifuged and dialyzed unconditioned media or secretomes were concentrated 10-fold (to 1.2 mL) using Amicon Ultra-15 3 kDa centrifugal filters (Millipore, Billerica, MA) at 4ºC. Immediately thereafter, 1 mL of concentrate was chromatographed on tandem Superdex 200 Increase - Superdex 75 10/300 GL (GE, Marlborough, MA) columns eluted in 50

mM ammonium bicarbonate (pH 8.5) at a flow-rate of 0.5 mL/min using Pharmacia FPLC system (Amersham Life Sciences, Uppsala, Sweden). One milliliter fractions were collected and 700 µL was lyophilized for western blot. The remaining 300 µL was aliquoted and stored at -80ºC prior to analyses using tau or Aβ immunoassays. Lyophilisates of SEC fractions were resuspended in 15 µL 1x tricine sample buffer, boiled at 100ºC for 10 min and electrophoresed on 10-20% polyacrylamide tricine gels (ThermoFisher, Waltham, MA). Proteins were transferred onto 0.2 □ M nitrocellulose and the filters boiled in 50 mL PBS and then incubated with the following antibodies overnight: 6E10 (1 µg/mL), or Tau5 (1 µg/mL) and K9JA (1 µg/mL). The following day, blots were washed, incubated with infrared-labeled secondary antibodies (1:17,000; LiCor Biosciences, Lincoln, NE) and imaged using a LiCor Odyssey scanner (LiCor Biosciences, Lincoln, NE).

Antibodies

The antibodies used and their sources are described in Table S2.

Data analysis

All statistical analyses of LTP were conducted in Prism v. 6.07 (GraphPad Software Inc., La Jolla, CA). The magnitude of LTP is expressed as the percentage of pre-HFS baseline EPSP amplitude (± SEM). The n refers to the number of animals per group. Control experiments were interleaved randomly throughout. For time-line graphical representation, EPSP amplitudes were grouped into 5-min epochs; for statistical analysis, EPSP amplitudes were grouped into 10-min epochs. One-way ANOVA with Sidak's multiple comparison test (one-way ANOVA-Sidak) was used to compare between groups of three or more. Two-way ANOVA with repeated measures with Sidak's multiple comparison test (two-way ANOVA RM-Sidak) was used when there were only two groups. Paired *t* tests were carried out to compare pre- and post-HFS values within groups. A value of $P < 0.05$ was considered statistically significant.

SUPPLEMENTAL REFERENCES

Carmel, G., Mager, E.M., Binder, L.I., and Kuret, J. (1996). The structural basis of monoclonal antibody Alz50's selectivity for Alzheimer's disease pathology. J Biol Chem *271*, 32789-32795.

Chang, E.A., Beyhan, Z., Yoo, M.S., Siripattarapravat, K., Ko, T., Lookingland, K.J., Madhukar, B.V., and Cibelli, J.B. (2010). Increased cellular turnover in response to fluoxetine in neuronal precursors derived from human embryonic stem cells. Int J Dev Biol *54*, 707-715.

Ghoshal, N., Garcia-Sierra, F., Wuu, J., Leurgans, S., Bennett, D.A., Berry, R.W., and Binder, L.I. (2002). Tau conformational changes correspond to impairments of episodic memory in mild cognitive impairment and Alzheimer's disease. Exp Neurol *177*, 475-493.

Hu, N.W., Nicoll, A.J., Zhang, D., Mably, A.J., O'Malley, T., Purro, S.A., Terry, C., Collinge, J., Walsh, D.M., and Rowan, M.J. (2014). mGlu5 receptors and cellular prion protein mediate amyloid-beta-facilitated synaptic long-term depression in vivo. Nat Commun *5*, 3374.

Israel, M.A., Yuan, S.H., Bardy, C., Reyna, S.M., Mu, Y., Herrera, C., Hefferan, M.P., Van Gorp, S., Nazor, K.L., Boscolo, F.S.*, et al.* (2012). Probing sporadic and familial Alzheimer's disease using induced pluripotent stem cells. Nature *482*, 216-220.

Jiang, P., Lagenaur, C.F., and Narayanan, V. (1999). Integrin-associated protein is a ligand for the P84 neural adhesion molecule. J Biol Chem *274*, 559-562.

Johnson-Wood, K., Lee, M., Motter, R., Hu, K., Gordon, G., Barbour, R., Khan, K., Gordon, M., Tan, H., Games, D.*, et al.* (1997). Amyloid precursor protein processing and A beta42 deposition in a transgenic mouse model of Alzheimer disease. Proc Natl Acad Sci U S A *94*, 1550-1555.

Kanmert, D., Cantlon, A., Muratore, C.R., Jin, M., O'Malley, T.T., Lee, G., Young-Pearse, T.L., Selkoe, D.J., and Walsh, D.M. (2015). C-terminally truncated forms of tau, but not full-length tau or its C-terminal fragments, are released from neurons independently of cell death. J Neurosci *35*, 10851-10865.

Kim, K.S., Miller, D.L., Chen, C.-M.J., Sapienza, V.J., Bai, C., Grundke-Iqbal, I., Currie, J.R., and Wisniewski, H.M. (1988). Production and characterization of monoclonal antibodies reactive to synthetic cerebrovascular amyloid peptide. Neurosci Res Commun *2*, 121-130.

Mably, A.J., Liu, W., Mc Donald, J.M., Dodart, J.C., Bard, F., Lemere, C.A., O'Nuallain, B., and Walsh, D.M. (2015). Anti-Abeta antibodies incapable of reducing cerebral Abeta oligomers fail to attenuate spatial reference memory deficits in J20 mice. Neurobiol Dis *82*, 372-384.

McDonald, J.M., Cairns, N.J., Taylor-Reinwald, L., Holtzman, D., and Walsh, D.M. (2012). The levels of water-soluble and triton-soluble Abeta are increased in Alzheimer's disease brain. Brain Res *1450*, 138-147.

Mercken, M., Vandermeeren, M., Lubke, U., Six, J., Boons, J., Van de Voorde, A., Martin, J.J., and Gheuens, J. (1992). Monoclonal antibodies with selective specificity for Alzheimer Tau are directed against phosphatase-sensitive epitopes. Acta Neuropathol *84*, 265-272.

Moore, S., Evans, L.D., Andersson, T., Portelius, E., Smith, J., Dias, T.B., Saurat, N., McGlade, A., Kirwan, P., Blennow, K.*, et al.* (2015). APP metabolism regulates tau proteostasis in human cerebral cortex neurons. Cell Rep *11*, 689-696.

Pankiewicz, J., Prelli, F., Sy, M.S., Kascsak, R.J., Kascsak, R.B., Spinner, D.S., Carp, R.I., Meeker, H.C., Sadowski, M., and Wisniewski, T. (2006). Clearance and prevention of prion infection in cell culture by anti-PrP antibodies. Eur J Neurosci *23*, 2635-2647.

Park, I.H., Arora, N., Huo, H., Maherali, N., Ahfeldt, T., Shimamura, A., Lensch, M.W., Cowan, C., Hochedlinger, K., and Daley, G.Q. (2008). Disease-specific induced pluripotent stem cells. Cell *134*, 877-886.

Saurat, N.G., Livesey, F.J., and Moore, S. (2016). Cortical Differentiation of Human Pluripotent Cells for In Vitro Modeling of Alzheimer's Disease. Methods Mol Biol *1303*, 267-278.

Shi, Y., Kirwan, P., and Livesey, F.J. (2012a). Directed differentiation of human pluripotent stem cells to cerebral cortex neurons and neural networks. Nat Protoc *7*, 1836-1846.

Shi, Y., Kirwan, P., Smith, J., Robinson, H.P., and Livesey, F.J. (2012c). Human cerebral cortex development from pluripotent stem cells to functional excitatory synapses. Nat Neurosci *15*, 477-486.

Wang, Y.P., Biernat, J., Pickhardt, M., Mandelkow, E., and Mandelkow, E.M. (2007). Stepwise proteolysis liberates tau fragments that nucleate the Alzheimer-like aggregation of full-length tau in a neuronal cell model. Proc Natl Acad Sci U S A *104*, 10252-10257.

Supplemental Table S1, Related to Experimental Procedures and main text.
List of Abbreviations

Abbreviation	Meaning
aCSF	artificial cerebrospinal fluid
AD	Alzheimer's disease
APP	amyloid precursor protein
APP^{Dp}	amyloid precursor protein gene duplications
APP^{Dp}.B	amyloid precursor protein gene duplications line B
APPsα	soluble APP-alpha
Aβ	amyloid β-protein
Aη	aη fragment of APP
A$\eta\alpha$	Soluble A$\eta\alpha$ fragment of APP
CT	C-terminal
ELISA	enzyme-linked immunosorbent assay
fAD	familial Alzheimer's disease
GFAP	glial fibrillary acidic protein
FL	full-length
i.c.v.	intracerebroventricular
ID	immunodepleted
iPSC	induced pluripotent stem cell
LTP	long-term potentiation
mAb	monoclonal antibody
NDC	non-demented control
NDC.B	non-demented control line B
MAP2	microtubule-associated protein 2
MR	mid-region
MTBR	microtubule-binding region
NT	N-terminal
NTE-Aβ	N-terminally extended Aβ
PAG	protein G Agarose beads
PAS	protein A Sepharose beads
PIS	preimmune serum
PrP	*prion protein*
PS1 Int4	mutation in *PSEN1* L113_I114insT

PS1 Int4.B	mutation in *PSEN1* L113_I114insT line B
rpm	revolutions per minute
RT	room temperature
SEC	size exclusion chromatography
TBR1	the neuron-specific transcription factor T-box brain 1
TBS	tris-buffered saline
TBST	tris-buffered saline containing 0.05% Tween 20
Ts21	mutation in trisomy of chromosome 21
Ts21.B	mutation in trisomy of chromosome 21 line B

Supplemental Table S2, Related to Experimental Procedures.
Primary antibodies and their antigens, dilutions and sources.

Antibody	Clonality	Antigen/Epitope	Dilution	Source	Reference
AW7	Polyclonal	Pan-Aβ	1:50 (IP)	Walsh Lab	(McDonald et al., 2012)
6E10	Monoclonal	702-706 of APP_{770}	1 µg/ml (WB); (i.c.v. injection)	Biolegend	(Kim et al., 1988)
266	Monoclonal	Aβ13-26	1 µg/ml (ELISA)	Janssen	(Johnson-Wood et al., 1997)
2G3	Monoclonal	Aβ terminating at Val40	1 µg/ml (ELISA)	Janssen	(Johnson-Wood et al., 1997)
21F12	Monoclonal	Aβ terminating at Ile42	1 µg/ml (ELISA)	Janssen	(Johnson-Wood et al., 1997)
Tau12	Monoclonal	Tau 6-18	2.5 µg/ml (ELISA)	Millipore	(Ghoshal et al., 2002)
BT2	Monoclonal	Tau 194-198	2.5 µg/ml (ELISA)	ThermoFisher	(Mercken et al., 1992)
Tau5	Monoclonal	Tau 210-241	1 µg/ml (WB); 1:100 (IP); 2.5 µg/ml (ELISA)	BioLegend	(Carmel et al., 1996)
K9JA	Polyclonal	Tau 243-441	1 µg/ml (WB); 2.5 µg/ml (ELISA)	Dako	(Wang et al., 2007)
TauAB	Monoclonal	Tau 425-441	2.5 µg/ml (ELISA)	MedImmune	Gift from A. Billinton/ M. Perkinton
46-4	Monoclonal	Anti-HIV	1:100 (IP)	ATCC	(Wang et al., 2007)
Anti-TBR1	Polyclonal	TBR1 50-150	1:1500 (ICC)	Abcam	(Shi et al., 2012c)
Anti-MAP2	Polyclonal	Full length MAP2	1:2000 (ICC)	Abcam	(Shi et al., 2012c)
Anti- β3-tubulin	Monoclonal	C-terminus of β3-tubulin	1:1000 (WB)	Biolegend	(Moore et al., 2015)
GFAP	Polyclonal	Full length GFAP	1:1000 (WB)	Abcam	(Chang et al., 2010)
Anti- β-actin	Monoclonal	N-terminus of β-actin	1:10000 (WB)	Sigma	(Moore et al., 2015)
6D11	Monoclonal	PrP 93-109	(i.c.v. injection)	BioLegend	(Pankiewicz et al., 2006)
IgG2a	Monoclonal	N/A	(i.c.v. injection)	BioLegend	(Jiang et al., 1999)

Supplemental Table S3, Related to Figure 3.
Summary of the tau species detected by ELISAs in iPSC-derived cortical neuron secretomes after mock or AW7 immunodepletion.

	NDC		Ts21		APP^{Dp}		PS1 Int4	
	**Mock ID**	**AW7 ID**	**Mock ID**	**AW7 ID**	**Mock ID**	**AW7 ID**	**Mock ID**	**AW7 ID**
**Full-length**	488.3 ± 0.6	266.7 ± 4.7	403.7 ± 7.2	737.1 ± 2.4	700.2 ± 18.8	1081.5 ± 21.9	724.7 ± 14.3	2,085.8 ± 20.2
**N-terminal**	4,574.3 ± 438.3	4,849.7 ± 91.3	1,873.7 ± 430.3	2,406.4 ± 111.3	8,362.5 ± 684.5	9,662.9 ± 288.0	4,101.058 ± 84.7	6,549.2 ± 461.0
**Mid-region**	21,378.0 ± 682.8	21,137.8 ± 582.9	6,956.4 ± 578.6	8,352.7 ± 302.9	44,597.8 ± 28.2	42,967.1 ± 545.8	15,747.4 ± 113.8	16,811.5 ± 479.1
**C-terminal 1**	1,730.8 ±127.8	1,781.5 ± 72.8	1,089.1 ± 8.0	2,103.2 ± 122.6	3,033.7 ± 190.9	3,869.3 ± 119.7	2,170.0 ± 64.5	4,522.3 ± 61.9
**C-terminal 2**	502.4 ± 2.1	351.5 ± 3.7	438.0 ± 0.8	752.9 ± 18.3	631.4 ± 1.5	965.3 ± 5.5	800.1 ± 45.5	1,991.2 ±61.9

Key: Values represent mean ± SD in pg/ml of three technical replicates. ND = not detected. NDC = non-demented control secretome, Ts21 = Trisomy 21 secretome, APP^{Dp} = *APP* duplication secretome, PS1 Int4 = *PSEN1* intron 4 secretome. Mock ID = mock immunodepletion, AW7 ID = immunodepletion with AW7
